# Therapeutic potential of *Cola nitida* against dexamethasone- and scopolamine-induced cognitive and cardiac dysfunction

**DOI:** 10.3389/fnbeh.2026.1803200

**Published:** 2026-09-09

**Authors:** Lawrence Dayo Adedayo, Olugbenga S. Michael, Gideon Opeyemi Ayilara, Mayode Elizabeth Olumoroti, Olubayode Bamidele, Saminu Samaila, Ibtihel Dhaya, Nafisat Dasola Akorede, Patrick Oluwole Abolarin, Laurette N. Ofodile

**Affiliations:** 1 Neurophysiology Unit, Department of Human Physiology, College of Health Sciences, Federal University Wukari, Taraba, Nigeria; 2 Department of Physiology, College of Health Sciences, Bowen University, Iwo, Nigeria; 3 Department of Physiology, Faculty of Basic Medical Sciences, Adeleke University, Ede, Osun, Nigeria; 4 Physiology Programme, College of Health Sciences, Bowen University, Iwo, Nigeria; 5 Laboratory of Neurophysiology, Cellular Physiopathology and Biomolecule Valorization, Faculty of Sciences, University of Tunis El Manar, Tunis, Tunisia; 6 Department of Biochemistry, Faculty of Basic Sciences, Eko University of Medicine and Health Sciences, Lagos, Nigeria; 7 Department of Physiology, School of Basic Medical Sciences, Babcock University, Ilishan-Remo, Ogun, Nigeria; 8 Department of Biological Science, School of Science, Yaba College of Technology, Lagos, Nigeria

**Keywords:** cardiometabolic syndrome, cognitive dysfunction, *Cola nitida*, dexamethasone, scopolamine

## Abstract

**Background:**

Cognitive dysfunction and cardiometabolic disturbances frequently coexist in neurological and metabolic disorders. Chronic systemic inflammation in cardiometabolic syndrome may promote neuroinflammation, damaging neurons and synapses and impairing memory pathways. *Cola nitida* is a medicinal plant widely recognized for its stimulant and therapeutic properties, yet its neuroprotective and cardiometabolic effects remain underexplored. Objective: This study investigated the effects of methanol extract of *Cola nitida* (CN) in a mouse model of memory and metabolic dysfunction induced by scopolamine and dexamethasone.

**Methods:**

Male mice were randomly divided into six groups (*n* = 5): control (saline), scopolamine (3 mg/kg), scopolamine + dexamethasone (0.2 mg/kg), CN (50 mg/kg) + scopolamine, CN + scopolamine + dexamethasone, and donepezil (1 mg/kg) + scopolamine + dexamethasone. Treatments lasted 7 days, followed by behavioral testing (Morris Water Maze, Y-Maze). Biochemical analyses included acetylcholinesterase activity, oral glucose tolerance, lactate/LDH, oxidative stress markers, and lipid profiles. Hippocampal histology assessed neurodegeneration, while molecular docking evaluated CN phytochemicals against proteins linked to glucose metabolism, oxidative stress, and cholinergic signaling.

**Results:**

Co-administration of dexamethasone and scopolamine impaired memory, elevated AChE activity, fasting glucose, and oxidative stress in the brain and heart. CN treatment improved memory and glucose tolerance, reduced oxidative stress and AChE activity, and preserved hippocampal neuronal integrity. Docking revealed strong affinities of CN flavonoids and procyanidins for Akt, GLP-1 receptor, Keap1, AChE, and TYK2, implicating them in glucose regulation, redox balance, and cholinergic neurotransmission.

**Conclusion:**

These findings highlight the neuroprotective and cardiometabolic benefits of *Cola nitida* and support its therapeutic potential for cognitive and metabolic disorders.

## Graphical Abstract

**Figure:**
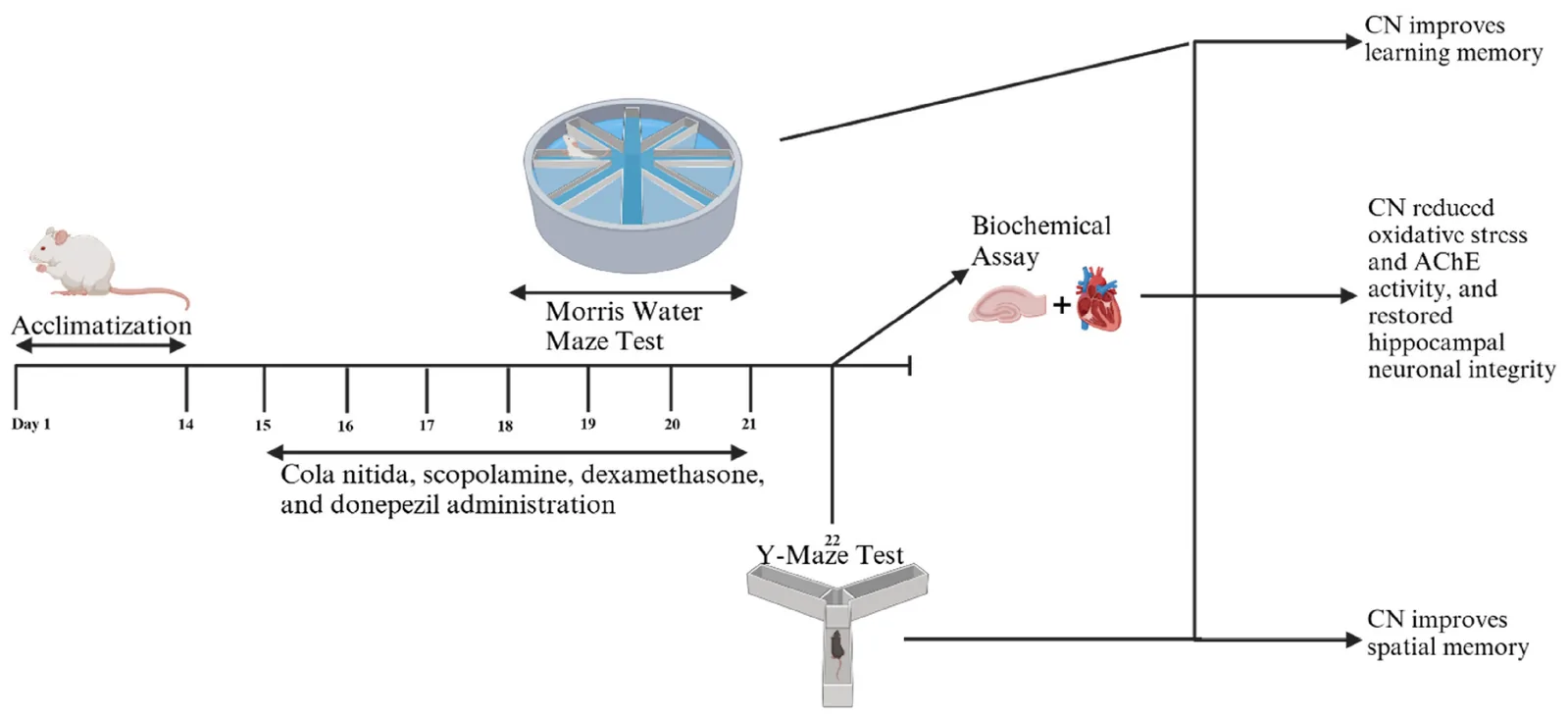
Summary of therapeutic potential of *Cola nitida* against dexamethasone- and scopolamine-induced cognitive and cardiac dysfunction.

## 1 Introduction

Cardiometabolic syndrome (CMS) is a multifaceted clinical disorder characterized by the interplay of cardiovascular disease, metabolic dysfunction, and risk factors for type 2 diabetes mellitus. It is typically defined by the coexistence of obesity, hypertension, dyslipidemia, and insulin resistance. Although sedentary lifestyles, unhealthy dietary habits, and hormonal imbalances are well-established contributors to its development, genetic predisposition plays a central role by interacting with these environmental and lifestyle factors (Anto et al., 2022). The prevalence of CMS varies across populations and geographic regions, but it remains particularly high among individuals with type 2 diabetes mellitus (Sakakibara et al., 2019).

The pathophysiology of CMS is characterized by interconnected mechanisms, including vascular dysfunction, insulin resistance, chronic inflammation, and persistent hypertension. Together, these pathological processes can impair the structure and function of brain regions involved in learning and memory, thereby contributing to cognitive decline and amnesia. Rojas et al. (2021) reported several CMS-associated neuropathological changes, including cerebral small vessel disease, tau hyperphosphorylation, and neuroinflammation. Clinically, these alterations manifest as memory impairment, mild cognitive impairment, and other neurocognitive disorders. Furthermore, insulin resistance disrupts insulin signaling within the brain, particularly in the hippocampus, leading to impaired synaptic plasticity and cognitive dysfunction. This brain-specific impairment in insulin signaling has been described as “type 3 diabetes” (Nguyen et al., 2020). Chronic systemic inflammation, a hallmark of CMS, can promote neuroinflammation, leading to neuronal and synaptic injury that disrupts memory-related neural circuits (Millán Solano et al., 2023). In addition, prolonged hypertension may contribute to hippocampal atrophy, further compromising memory function (Fiford et al., 2020). Consequently, CMS is recognized as a major risk factor for cognitive impairment and amnesia. The structural and functional brain alterations associated with CMS also increase the risk of both vascular dementia and Alzheimer’s disease (Lamar et al., 2020; Lecordier et al., 2021). Notably, memory impairment is one of the most common cognitive deficits observed in individuals with CMS, underscoring the substantial impact of the syndrome on cognitive health (Kouvari et al., 2022).

Donepezil, an acetylcholinesterase inhibitor, is widely used in the management of Alzheimer’s disease and vascular dementia. It improves cognitive function by inhibiting acetylcholinesterase, thereby increasing acetylcholine availability and enhancing central cholinergic neurotransmission. However, the increased availability of acetylcholine in peripheral tissues, particularly the heart, may augment parasympathetic activity and increase the risk of bradycardia, especially in susceptible individuals. Moreover, although donepezil provides symptomatic improvement in cognition and daily functioning, there is no convincing evidence that it modifies the underlying disease process or slows the progression of neurodegenerative disorders (Kumar et al., 2025). Current pharmacological therapies for metabolic syndrome have also been proposed to possess neuroprotective properties; however, the supporting evidence remains inconclusive, and some agents have even been associated with an increased risk of dementia (Moore et al., 2013; Thong et al., 2023).

Given the limitations of current pharmacological therapies, there is increasing interest in medicinal plants that have traditionally been used to enhance memory and manage metabolic disorders (Mehla et al., 2020; Sankar et al., 2025). One such plant is *Cola nitida*, a member of the Sterculiaceae family that is native to the tropical regions of West Africa. Traditionally, *C. nitida* has been used to relieve a variety of ailments, including asthma, bronchitis, headaches, toothache, gingival pain, peptic ulcers, and menstrual disorders (Dah-Nouvlessounon et al., 2015; Adeosun et al., 2017). The seeds (kola nuts) contain approximately 2% caffeine and are widely chewed for their stimulant effects, particularly to enhance alertness and reduce fatigue (Dah-Nouvlessounon et al., 2015). Beyond its traditional uses, accumulating experimental evidence indicates that *C. nitida* possesses a broad range of pharmacological activities, including antihyperglycemic, hypolipidemic, anti-arthritic, and cardioprotective effects (Ekalu and Habila, 2020). In addition, Adedayo et al. (2019) demonstrated that *C. nitida* exhibits potent antioxidant, anti-inflammatory, and analgesic properties, further supporting its therapeutic potential in the management of chronic metabolic and inflammatory disorders.

The therapeutic potential of *Cola nitida* in cognitive impairment and cardiometabolic disorders is largely attributed to its rich phytochemical composition, which includes flavonoids, L-epicatechin, D-catechin, and other phenolic compounds. These bioactive constituents possess antioxidant, anti-inflammatory, and metabolic regulatory properties that may protect against the pathological processes underlying both cognitive dysfunction and cardiometabolic syndrome. Therefore, the present study aimed to investigate the effects of the methanol extract of *C. nitida* on dexamethasone-induced cardiometabolic dysfunction and scopolamine-induced amnesia in male mice. Specifically, the study evaluated the effects of *C. nitida* on learning and memory and explored the biochemical mechanisms through which the extract exerts its neuroprotective and cardiometabolic effects.

A review of the literature revealed that most studies on *C. nitida* have been limited to *in vivo* and *in vitro* investigations, with little attention given to *in silico* or computational approaches. In particular, there is a paucity of computational studies examining the interactions between *C. nitida* phytochemicals and molecular targets involved in glucose metabolism, lipid homeostasis, oxidative stress, cholinergic neurotransmission, and neuroinflammation. To address this knowledge gap, molecular docking analyses were performed to evaluate the binding interactions of selected bioactive compounds from *C. nitida* with key protein targets implicated in glucose metabolism, oxidative stress, acetylcholinesterase activity, and neuroinflammatory signaling. These analyses were intended to provide mechanistic insights into the neuroprotective and cardiometabolic potential of *C. nitida*.

## 2 Materials and methods

### 2.1 Drugs and plant material

Scopolamine, Donepezil, Dexamethasone, Ketamine and Xylazine were purchased from Alpha Pharmacy, Ikeja, Lagos, and Consilient Health Ltd., Clon-Skeagh, Dublin, Ireland. Fresh seeds/nuts of *Cola nitida* were collected at the Odo Ori market in Iwo. The identification and authentication of the seeds were carried out at the Department of Botany, Obafemi Awolowo University, Ile-Ife, Nigeria, with voucher number: FHI. 17512. Preparation of the methanol extracts of the seeds was as described in Adedayo et al. (2019).

### 2.2 Animals

Thirty male Balb/c mice, weighing 20–30 g and aged 8–10 weeks, were obtained from University College Hospital in Ibadan, Oyo State. The animals were maintained under standard nutritional and environmental conditions, including normal relative humidity, room temperature, and a 12-h light/dark cycle throughout the study. The acclimatization period lasted 2 weeks, during which animals received standard feed and water *ad libitum*. The mice were housed at the experimental animal facility at the College of Health Sciences, Bowen University, Iwo, Osun State, in well-ventilated plastic cages with wire mesh covers. The study was approved by the Ethics Committee for Animal Experimentation of the College of Health Sciences, Bowen University (Approval no. BUTH/REC/377), and was conducted following the Guide for the Care and Use of Laboratory Animals published by the US National Institutes of Health (NIH Publication No. 85-23, revised 2011).

### 2.3 Experimental design

Animals were randomly assigned into six groups (*n* = 5) as follows (Table 1);

**TABLE 1:** Animal grouping.

Group	Treatment description
Group 1 (control)	Received 10 mL/kg distilled water orally.
Group 2 (scopolamine only)	Received 3 mg/kg scopolamine intraperitoneally (i.p.).
Group 3 (dexamethasone + scopolamine)	Received 0.2 mg/kg dexamethasone orally to induce metabolic dysfunction, followed 30 min later by 3 mg/kg scopolamine i.p.
Group 4 (*Cola nitida* + scopolamine)	Pre-treated with 50 mg/kg methanol extract of *Cola nitida*. One hour later, received 3 mg/kg scopolamine was administered i.p.
Group 5 (dexamethasone + scopolamine + *Cola nitida*)	Received 0.2 mg/kg dexamethasone orally. After 15 min, 3 mg/kg scopolamine was administered i.p., followed 30 min later by oral administration of 50 mg/kg *Cola nitida*.
Group 6 (dexamethasone + scopolamine + donepezil)	Received 0.2 mg/kg dexamethasone orally. After 15 min, 3 mg/kg scopolamine was administered i.p., followed 30 min later by 1 mg/kg donepezil administered orally.

All treatments scopolamine (3 mg/kg, i.p.), dexamethasone (0.2 mg/kg, oral), and *Cola nitida* extract (50 mg/kg, oral) were administered once daily for seven consecutive days. Rahimzadegan and (Rahimzadegan and Soodi, 2018) previously induced memory impairment using 3 mg/kg scopolamine intraperitoneal administration in rodents. Likewise, metabolic disorder was induced in rodents through oral administration of 0.2 mg/kg dexamethasone in studies such as Motta et al. (2018) and Caparroz-Assef et al. (2007). Likewise, Adedayo et al. (2019) showed the antioxidant, anti-inflammatory, and analgesic properties of *C. nitida* at 50 mg/kg. Group 4 served as preventive intervention while group 5 and 6 served as therapeutic intervention.

All experimental procedures adhered to the United States National Institute of Health guidelines for care and use of laboratory animals in Biomedical Research (National Research Council Committee for the Update of the Guide for the Care and Use of Laboratory Animals, 2011).

### 2.4 Behavioral assessment

The Morris Water Maze (MWM) test was employed to evaluate spatial learning and reference memory in mice. The water temperature was maintained at room temperature throughout the experiment to minimize stress and ensure uniform testing conditions. The circular pool was conceptually divided into four equal quadrants designated as North-East (NE), North-West (NW), South-East (SE), and South-West (SW). A circular escape platform measuring approximately 8 cm in diameter was positioned at the center of the North-East (NE) quadrant and submerged approximately 1 cm below the surface of the water. The water was made opaque by addition of non-toxic liquid milk. This served to obscure the submerged escape platform and eliminate visual cues beneath the water surface.

Mice were acclimatized to the testing environment to reduce anxiety and novelty-induced behavioral variations. During each trial, the mouse was gently released into the water facing the wall of the pool from the SW quadrant, which served as the designated starting point and was allowed to swim freely in search of the hidden platform. Each trial was terminated when the mouse successfully located and climbed onto the submerged platform. Mice that could not locate the escape platform within the duration of 60 s were gently guided to the platform by the investigator.

Upon completion of each trial, the mouse was carefully removed from the pool, gently dried using a clean absorbent towel to prevent hypothermia, and returned to its home cage. An inter-trial interval of approximately 15 min was maintained between successive trials to allow adequate recovery. Mice underwent three acquisition trials per day for a total period of 7 days. The escape latency, i.e., time taken to locate the hidden platform was recorded manually. Following completion of the acquisition phase, a probe trial was conducted on Day 8 to assess memory retention. During the probe trial, the escape platform was removed from the pool, and each mouse was released from the quadrant opposite the former platform location. The animal was allowed to swim freely for 60 s, during which memory performance was assessed by measuring the time spent in the target quadrant.

Spatial reference memory was assessed using a probe test and the Y-Maze apparatus. Alternations were defined as consecutive entries into all three arms, and spontaneous alternation percentage (SA%) was calculated. The number of maximum spontaneous alternation was calculated as the total number of arms entered minus 2, and the percentage was calculated as follows:


$$\%\text{Alternation}=\left(\frac{\mathrm{Number}\,\mathrm{of}\,\mathrm{Spontaneous}\,\mathrm{Alternations}}{\mathrm{Total}\,\mathrm{Arm}\,\mathrm{Entries}-2}\right)\times 100$$

### 2.5 Biochemical assessments

Serum biochemical indices were assessed using enzymatic colorimetric methods. Total cholesterol was quantified by enzymatic hydrolysis and oxidation, producing hydrogen peroxide that reacted with peroxidase to form a colored compound measured at 500 nm. Triglycerides were determined by hydrolysis to glycerol and subsequent oxidation with glycerol oxidase, with the resulting peroxide generating a measurable color in a peroxidase-catalyzed system. Uric acid levels were assayed using uricase, which oxidized uric acid to yield peroxide, subsequently forming a quinoneimine dye in the presence of peroxidase, with absorbance proportional to concentration. Malondialdehyde (MDA), an index of lipid peroxidation, was measured after protein precipitation and reaction of the supernatant with MDA-TBA salt under acidic conditions to generate MDA quantitatively.

Acetylcholinesterase, lactate dehydrogenase, Glutathione Reductase (GR), and reduced Glutathione (GSH) were determined using ElabScience Biotechnology Co., Ltd. (Wuhan, China) ELISA Kits.

### 2.6 Histological study

Two mice in each group were selected for a histology study. The animals were anesthetized by intraperitoneal administration of ketamine (75 mg/kg) and xylazine (5 mg/kg). The brains were perfused through a transcardial perfusion of 200 mL normal saline followed by 200 mL of 4% formaldehyde. The brains, hearts, and livers of the mice were harvested and preserved in 10% formalin. Hematoxylin and Eosin stain (H and E stain) and Nissl stain studies were carried out in the Department of Pathology, University College Hospital, Ibadan, to determine the morphological changes in the hippocampus. The results were interpreted by a neuropathologist in the Department of Pathology, University College Hospital (UCH), Ibadan.

### 2.7 Ligands preparation

The ligands were selected based on literature data reporting their biological relevance, as well as on the phytochemical composition of the C. nitida. According to the study by Akinola et al. (2020) the methanol extract of *Cola nitida* contains several bioactive compounds, including caffeine, catechin, epicatechin, naringin, procyanidin B1, procyanidin B2, and theobromine. In this study, all these compounds were subjected to molecular docking to evaluate their potential interactions with selected protein targets. The ligands simplified molecular input line entry system (SMILES) notations (Table 2) were retrieved from the PubChem database.^1^ Subsequently, their 3D conformations were generated and energy-minimized using Avogadro2^2^ in order to obtain the most stable structures. The optimized molecules were then exported as a protein databank file (.pdb) for further analysis.

**TABLE 2:** Molecular descriptors of *Cola nitida* phytochemicals used for docking simulations.

Compound	Pubchem ID	MW (g/moL)	Smiles
Caffeine	2519	194.19	CN1C=NC2=C1C(=O)N(C(=O)N2C)C
Catechin	9064	290.27	C1[C@@H]([C@H](OC2=CC(=CC(=C21)O)O)C3=CC(=C(C=C3)O)O)O
Epicatechin	72276	290.27	C1[C@H]([C@H](OC2=CC(=CC(=C21)O)O)C3=CC(=C(C=C3)O)O)O
Naringin	442428	580.5	C[C@H]1[C@@H]([C@H]([C@H]([C@@H](O1)O[C@@H]2[C@H]([C@@H]([C@H](O[C@H]2OC3 =CC(=C4C(=O)C[C@H](OC4=C3)C5=CC=C(C=C5)O)O)CO)O)O)O)O)O
Procyanidin B1	11250133	578.5	C1[C@@H]([C@H (OC2 = C1C( = CC( = C2[C@@H]3[C@H]([C@H](OC4 = CC( = CC( = C34)O)O)C5 = CC( = C(C = C5)O)O)O)O)O)C6 = CC( = C(C = C6)O)O)O
Procyanidin B2	122738	578.5	C1[C@H]([C@H (OC2 = C1C( = CC( = C2[C@@H]3[C@H]([C@H](OC4 = CC( = CC( = C34)O)O)C5 = CC( = C(C = C5)O)O)O)O)O)C6 = CC( = C(C = C6)O)O)O
Theobromine	5429	180.16	CN1C = NC2 = C1C( = O)NC( = O)N2C

The crystallographic structures of murine target proteins were retrieved from Protein Data Bank.^3^

The selected protein targets were chosen based on their well-established involvement in glucose metabolism, cholinergic activity, oxidative stress, and memory impairment. Proteins involved in glucose metabolism, such as Protein kinase B (Akt) (PDB ID: 3OW3, 1.9A°) and Glucagon-like peptide-1 receptor (PDB ID: 6XOX, 3.1A°) were used in this study. For the Acetylcholinesterase (AChE) activity, murine AChE structure (PDB ID: 8ORC, 2.1A°) was chosen. To investigate the oxidative stress-related pathway, the Kelch-like ECH-associated protein 1 (Keap1) domain (PDB ID: 7OFE, 1.19A°) was selected. Regarding memory impairment, the tyrosine-protein kinase 2 (TYK2) (PDB ID: 4E20, 2.6A°) was selected due to its essential role in the phosphorylation of the signal transducer and activator of transcription 1 (STAT1).

### 2.8 Molecular docking

**FIGURE 1:**
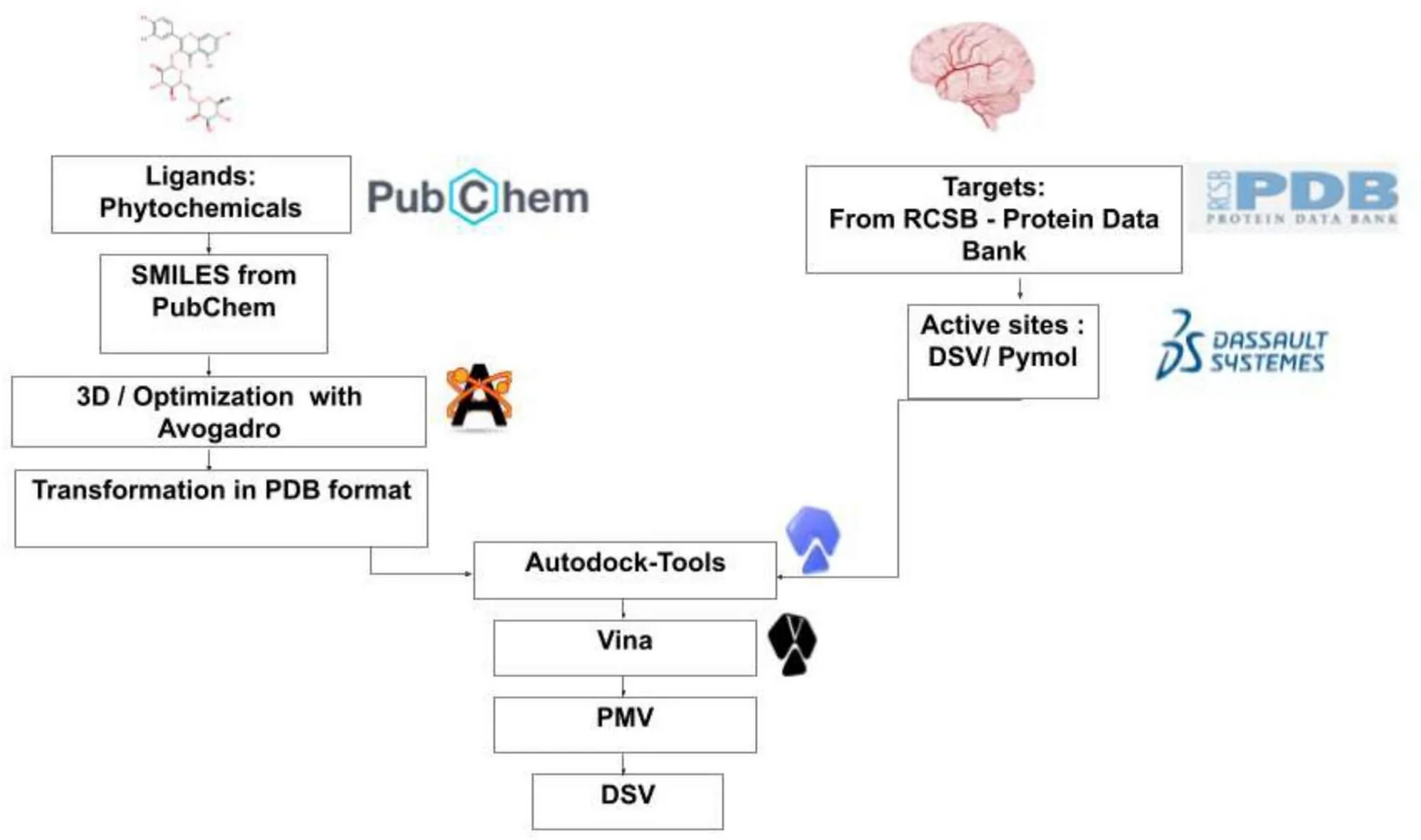
*In silico* workflow.

Molecular docking was performed using the Autodock suite engine MGLTools 1.5.6 package (Trott and Olson, 2010; The Scripps Research Institute, 2014), which includes the Graphical User Interface AutodockTools, a module to generate docking input files, PMV (Python Molecular Viewer) that allows creation of resulting conformations at the end of docking process and Autodock vina 1.2 (Eberhardt et al., 2021) that performs docking (Figure 1).

Using AutoDockTools, missing atoms were corrected, polar hydrogens were added, and Kollman partial atomic charges were assigned to the proteins. For the ligands, Gasteiger charges were applied, and their rotatable bonds were defined to allow flexibility, while the protein targets were kept rigid during docking.

Targeted docking was applied for all protein targets, as the active binding sites were experimentally defined by the presence of co-crystallized ligands. The docking grids were centered on these sites following visual inspection and validation using Discovery Studio Visualizer (DSV; Dassault Systèmes, version 2017.R2, San Diego), ensuring consistency and reliability of the docking protocol and allowing a focused and biologically relevant analysis. The grid box parameters employed in the docking simulations are summarized in Table 3.

**TABLE 3:** Molecular docking parameters.

Protein	ID	Binding site	Grid parameters	RMSD score (A°)	References
Akt	3OW3	**A:** LEU49, GLY50, THR51, GLY52, VAL57, ALA70, LYS72, VAL104, MET120, GLU121, TYR122, VAL123, GLU127, LEU173, THR183, ASP184, PHE327, HOH669, HOH836 **B:** ARG18	Center_x = 7.602 center_y = 32.067 center_z = 36.006 size_x, y, z = 40,40,40	1.629	(Bencsik et al., 2010)
Glucagon-like peptide-1 receptor (GLP-1R)	6XOX	**R:** SER31, TRP33, GLU34, PRO137, GLU138, LEU141, LEU144, TYR145, TYR148, LYS197 ASP198, ALA200, LEU201, LYS202, MET204, TYR205, TYR220, CYS226, VAL229, PHE230, MET233, THR298, LEU384, LEU388	Center_x = 152.464 center_y = 143.939 center_z = 119.455 size_x, y, z = 40,40,40	0.218	(Kawai et al., 2020)
Acetylcholinesterase	8ORC	**A:** ASP74, TPR86, GLY120, GLY121, GLY122, TYR124, SRE125, TYR133, GLU202, SER203, TRP286, PHE297, TYR337, PHE338, TYR341, HIS447, GLY448, TYR449, HOH732, HOH742, HOH775, HOH920	Center_x = 28.671 center_y = 20.776 center_z = 12.586 size_x, y, z = 40,40,40	0.617	(Kumar et al., 2025)
Keap1 Kelch domain	7OFE	**A:** TYR334, ARG415, ARG483, SER508, GLY509, TYR525, GLN530, SER555, ALA556, TYR572, PHE577, SER602, HOH975	Center_x = -43.100 center_y = 20.768 center_z = -9.869 size_x, y, z = 030,30,30	0.104	(Narayanan et al., 2022)
Tyrosine-protein kinase TYK2	4E20	**A:** LEU899, GLY900, GLU901, GLY902, GLY 905, LYS906, VAL907, ALA924, LYS926, VAL965, MET974, TYR976, VAL977, GLY980, ASP1037, ARG 1023, ASN1024, LEU1026, GLY1036, ASP1037, HOH1329, HOH1350	Center_x = 32.404 center_y = -10.831 center_z = 26.933 size_x, y, z = 40,40,40	0.106	(Argiriadi et al., 2012)

Molecular docking was carried out using AutoDock Vina due to its validated scoring function, computational efficiency, and widespread use in structure-based drug design. The estimated free energy of binding was calculated with an exhaustiveness of 32, and the top ten binding poses within 4 kcal/mol of the best-scoring pose were retained for analysis.

The docking results were processed and visualized with PMV. The best-docked pose, defined as the conformation with the lowest binding energy, was selected and visualized using Biovia Discovery Studio Visualizer.

Docking protocol validation was performed using a redocking approach. The co-crystallized ligand was extracted from the experimentally resolved protein–ligand complex using PyMOL v0.99, and the corresponding ligand-free receptor structure was prepared for redocking. The native ligand was subsequently re-docked into its original binding site using AutoDock Vina under the same docking parameters applied for the tested compounds. The accuracy of the docking protocol was assessed by comparing the predicted redocked pose with the experimentally determined crystallographic conformation. The RMSD values between the two ligand poses were calculated using PyMOL v0.99, and values below 2.0 Å were considered indicative of a reliable docking protocol. The obtained RMSD values confirming the validity of the docking procedure are presented in Table 3.

### 2.9 Statistical analysis

The data were analyzed using GraphPad Prism Software version 5.0 and were expressed as Mean ± Standard error of the mean (SEM). Statistical analysis was done using ANOVA analysis, followed by Newman-Keuls *post-hoc* statistical tests. Comparison between the control and the treated groups was made, and values of *p* < 0.05 were considered significant. Comparison between the scopolamine and the treated groups was also made, and values of *p* < 0.05 were considered significant. The observed power for the sample size was 91%.

## 3 Results

### 3.1 Effects of methanol extract of *Cola nitida* administration on animal weight

Table 4 shows the effect of the methanol extract of *Cola nitida* (MECN) in weight in mice using *Cola nitida* (50 mg/kg), Scopolamine (3 mg/kg), DEX (0.2 mg/kg), and Donepezil (1 mg/kg).

**TABLE 4:** Effects of MECN administration on animal weight.

Group	Initial weight (g)	Final weight (g)	%Change in weight(%)
Control	24.3 ± 0.64	23.3 ± 1.37	-4.11523
SC	23.8 ± 0.86	23.3 ± 1.24	-2.10084
DEX + SC	24.3 ± 0.90	22.6 ± 0.73	-6.99588
CN + SC	23.9 ± 1.04	23.9 ± 2.00	0 *^#^
DEX + SC + CN	23.9 ± 0.77	25.3 ± 1.25	5.857741
DEX+SC+DP	23.9 ± 0.67	22.3 ± 1.93	-6.69456

The results are expressed as means ± SEM (*n* = 5). Data were analyzed using One-way ANOVA followed by Newman-Keuls’ *post-hoc* test. *p < 0.05 compared with control. ^#^*p* < 0.05 compared with SC group.

CN ameliorates the effect of body weight reduction induced by SC. There was a negative percentage weight change in SC (3 mg/kg) treated group and this was brought to normal in both CN+SC group and DEX+SC+CN group.

### 3.2 Behavioral assessments

#### 3.2.1 Effect of methanolic extract of CN on the total quadrant time of the animals in Morris Water Maze model

**FIGURE 2:**
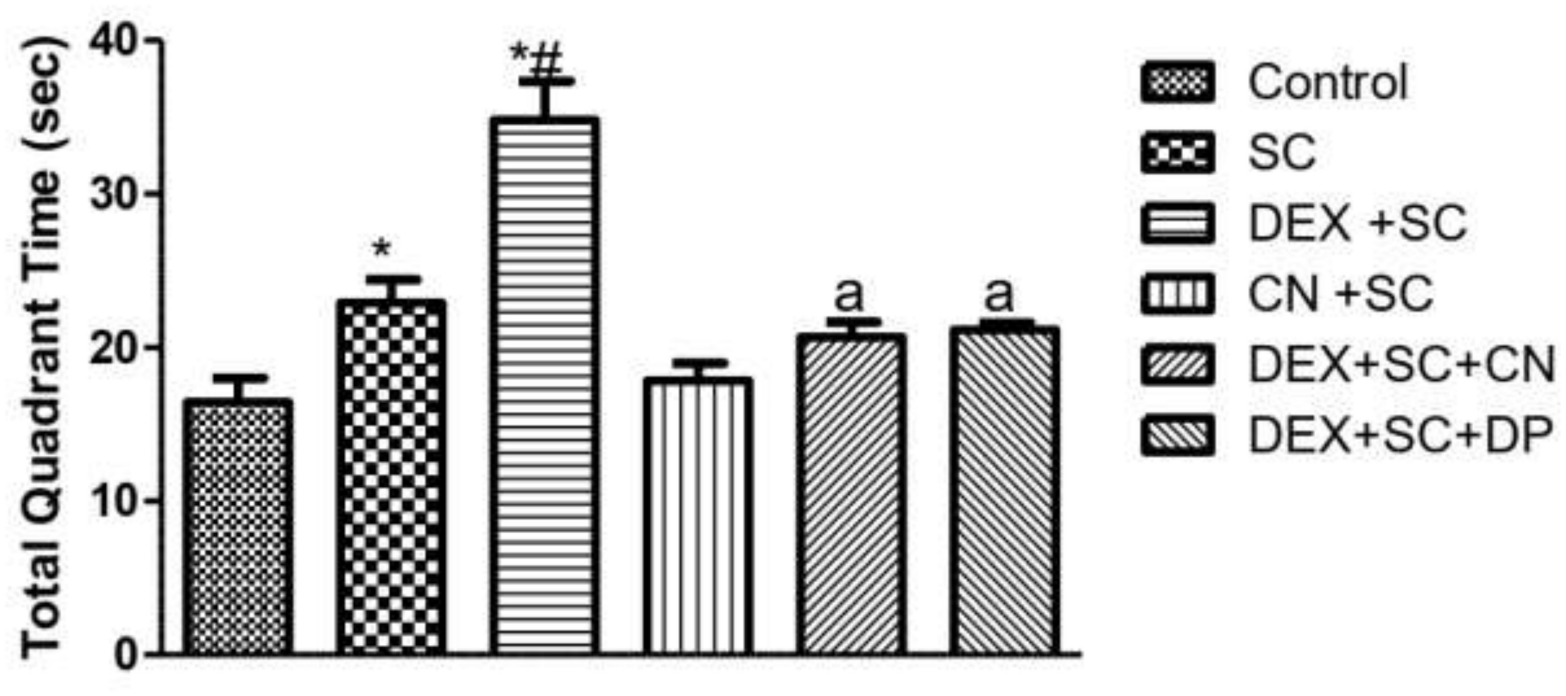
Effect of methanolic extract of CN on the total quadrant time (TQT) of the animals in Morris Water Maze model. Comparison bars indicate significant differences. *Significant difference from control group at *p* < 0.05. ^#^Significant difference from SC group at *p* < 0.05. ^a^Significant difference from the DEX+SC group. The results are expressed as means ± SEM (*n* = 5). Data were analyzed using One-way ANOVA followed by Newman-Keuls’ *post-hoc* test.

The result in Figure 2 shows the total quadrant time (TQT) of the animals in the Morris Water Maze model. There was a significant increase (*p* < 0.05) in total quadrant time in the SC and DEX+SC groups when compared with the control. Additionally, there was significant increase (*p* < 0.05) in the total quadrant time in the DEX+SC group when compared with SC group. Administration of *Cola nitida* and DP significantly reduced (*p* < 0.05) total quadrant time when compared with DEX+SC group.

#### 3.2.2 Effect of methanol extract of *Cola nitida* on escape latency time (ELT) in Morris Water Maze

**FIGURE 3:**
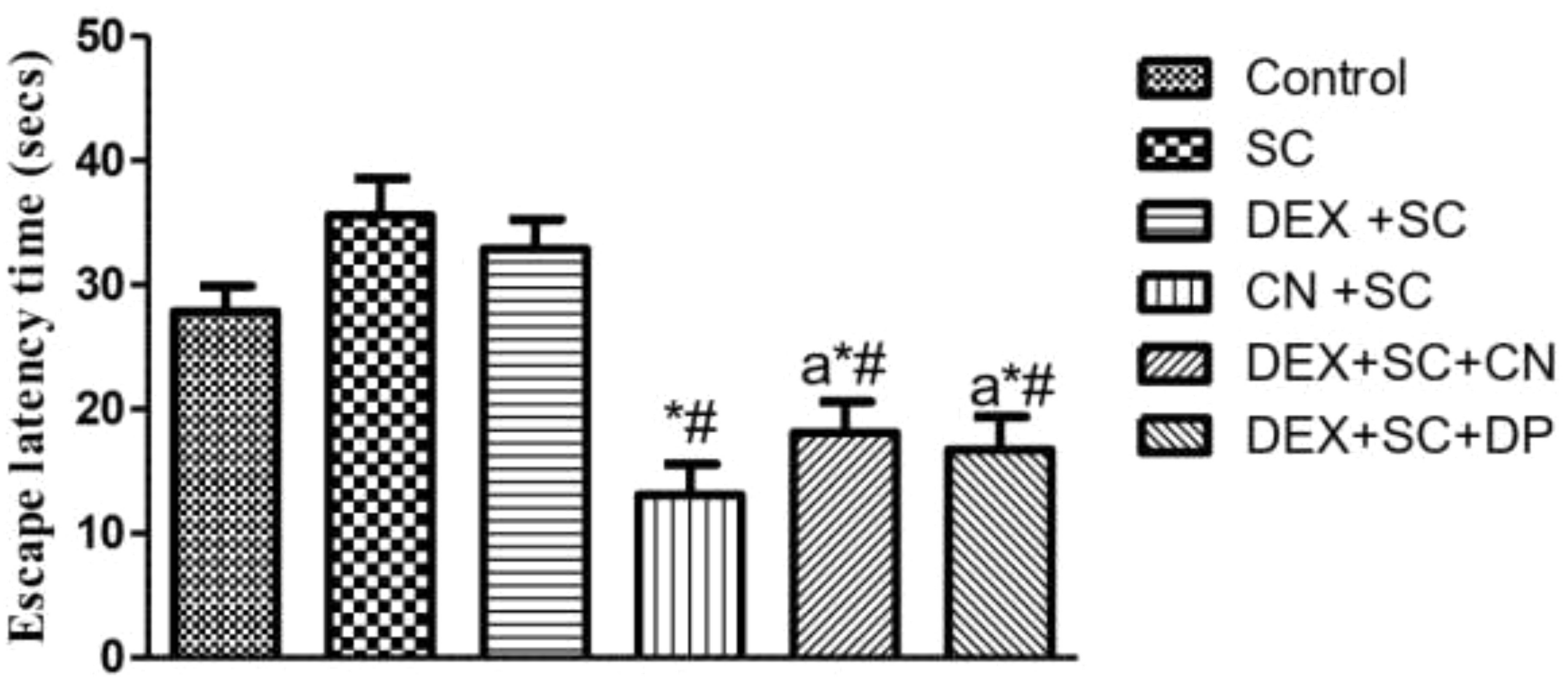
Effect of methanol extract of *Cola nitida* on escape latency time (ELT) in Morris Water Maze. Comparison bars indicate significant differences. *Significant difference from control group at *p* < 0.05. ^#^Significant difference from SC group at *p* < 0.05. ^a^Significant difference from the DEX+SC group. The results are expressed as means ± SEM (*n* = 5). Data were analyzed using One-way ANOVA followed by Newman-Keuls’ *post-hoc* test.

Figure 3 shows the Escape latency time (ELT) of the animals in Morris Water Maze model. There was a significant decrease (*p* < 0.05) in ELT in CN + SC, DEX + SC + CN, and DEX + SC + DP groups compared with control, SC, and DEX + SC groups.

#### 3.2.3 Effect of methanol extract of *Cola nitida* on time spent in quadrant in Morris Water Maze on probe day trial

**FIGURE 4:**
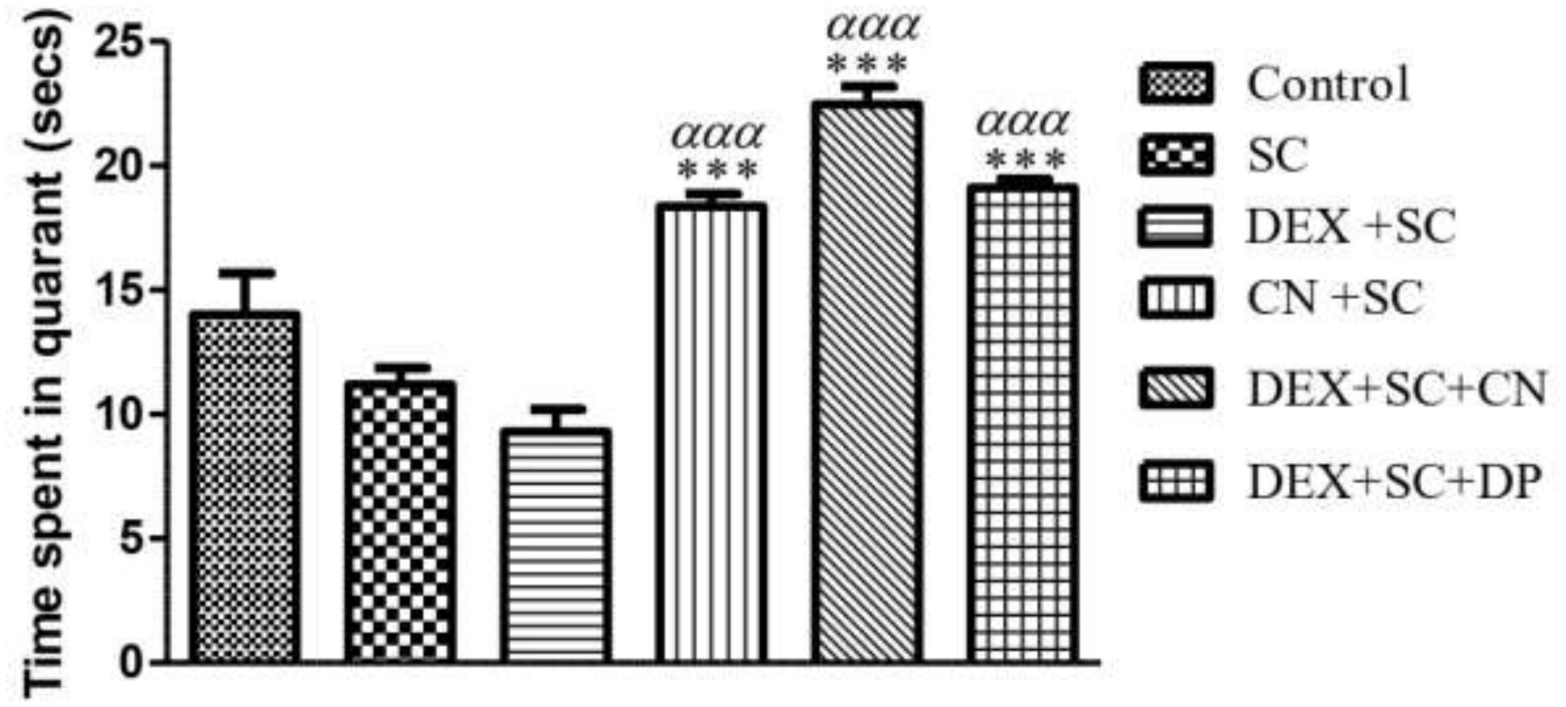
Effect of methanol extract of *Cola nitida* on time spent in quadrant in Morris Water Maze on probe day trial. Comparison bars indicate significant differences. ***Significant difference from control group at *p* < 0.001. *^aaa^*Significant difference from the SC group at *p* < 0.001. The results are expressed as means ± SEM (*n* = 5). Data were analyzed using one-way ANOVA followed by Newman-Keuls’ *post-hoc* test.

The result in Figure 4 shows the probe test of the animals in Morris Water Maze model. There was a significant increase (*p* < 0.001) in Time spent in Quadrant in CN + SC, DEX + SC + CN, and DEX + SC + DP groups when compared with control and SC groups on probe day trial.

#### 3.2.4 Effect of methanol extract of *Cola nitida* on % of alternation in Y-Maze

**FIGURE 5:**
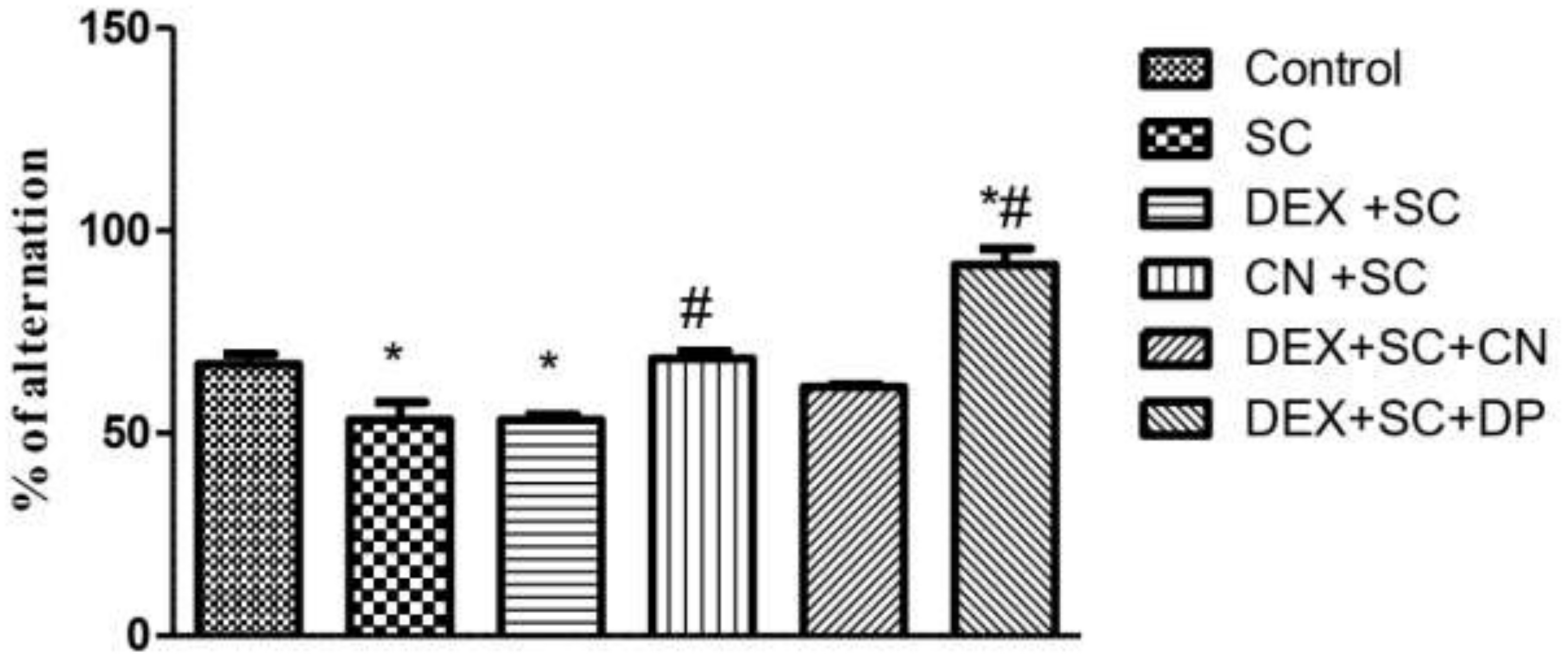
Effect of methanol extract of *Cola nitida* on % of alternation in Y Maze. Comparison bars indicate significant differences. *Significant difference from control group at *p* < 0.05. #Significant difference from SC group at *p* < 0.05. The results are expressed as means ± SEM (*n* = 5). Data were analyzed using One-way ANOVA followed by Newman-Keuls’ *post-hoc* test.

The result in Figure 5 shows the percentage alternation in Y-Maze model. Administration of SC and DEX significantly reduce (*p* < 0.05) compared to the control group. However, there was significant increase (*p* < 0.05) in percentage alternation in CN + SC and DEX + SC + DP groups when compared to SC group.

### 3.3 Histological studies

**FIGURE 6:**
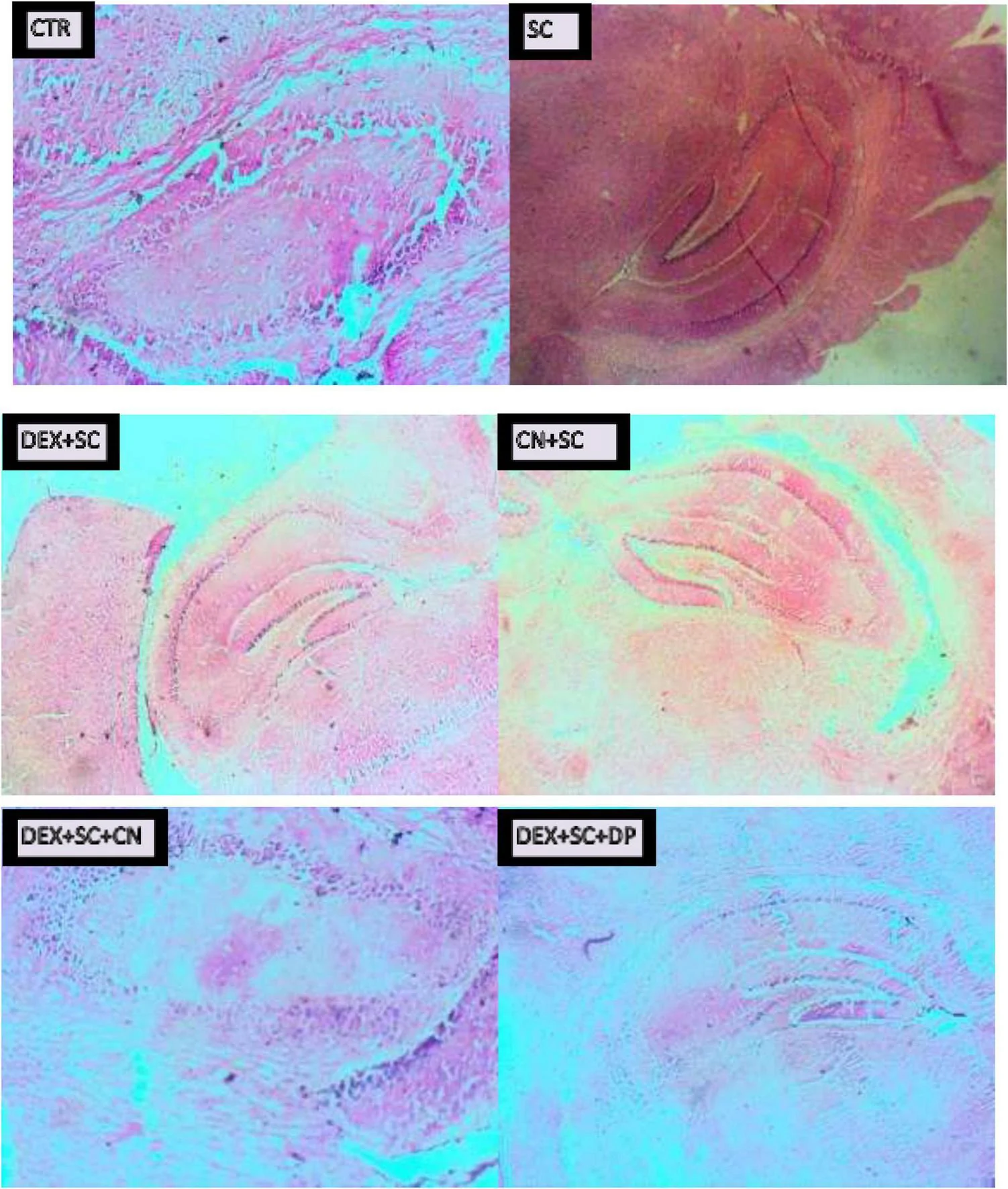
Representative photomicrographs of the effect of methanol extract of *Cola nitida* on the hippocampal region of mice stain with H and E. The control group has normal morphological organization of the cornu ammonis and normal neuronal cells. SC group shows coagulation necrosis and pyknosis of the pyramidal neurons. Neuronal degeneration, gliosis, and pyknosis were observed in the CA1 of DEX+SC group. Normal morphological organization of normal neuronal cells are seen in the CN+SC and DEX+SC+DP groups. H&E Mag × 400.

**FIGURE 7:**
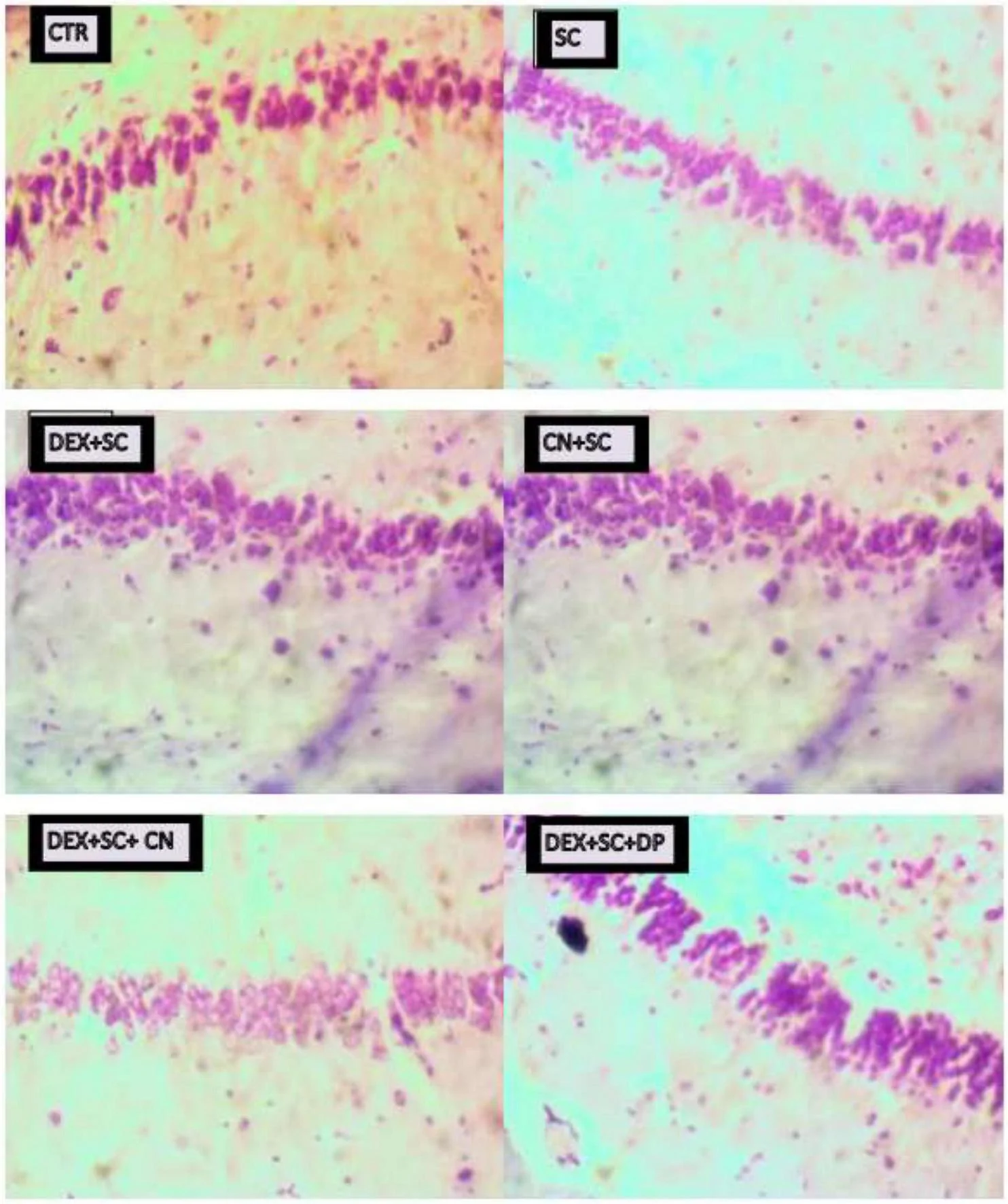
Representative photomicrographs of the effect of methanol extract *of Cola nitida* on hippocampal area CA2 of mice. The control group has normal morphological organization of the cornu ammonis and normal neuronal cells. SC group shows coagulation necrosis and pyknosis of the pyramidal neurons. Normal morphological organization of normal neuronal cells are seen in the CN+SC, DEX+SC+CN and DEX+SC+DP groups. Nissl Stain × 400.

**FIGURE 8:**
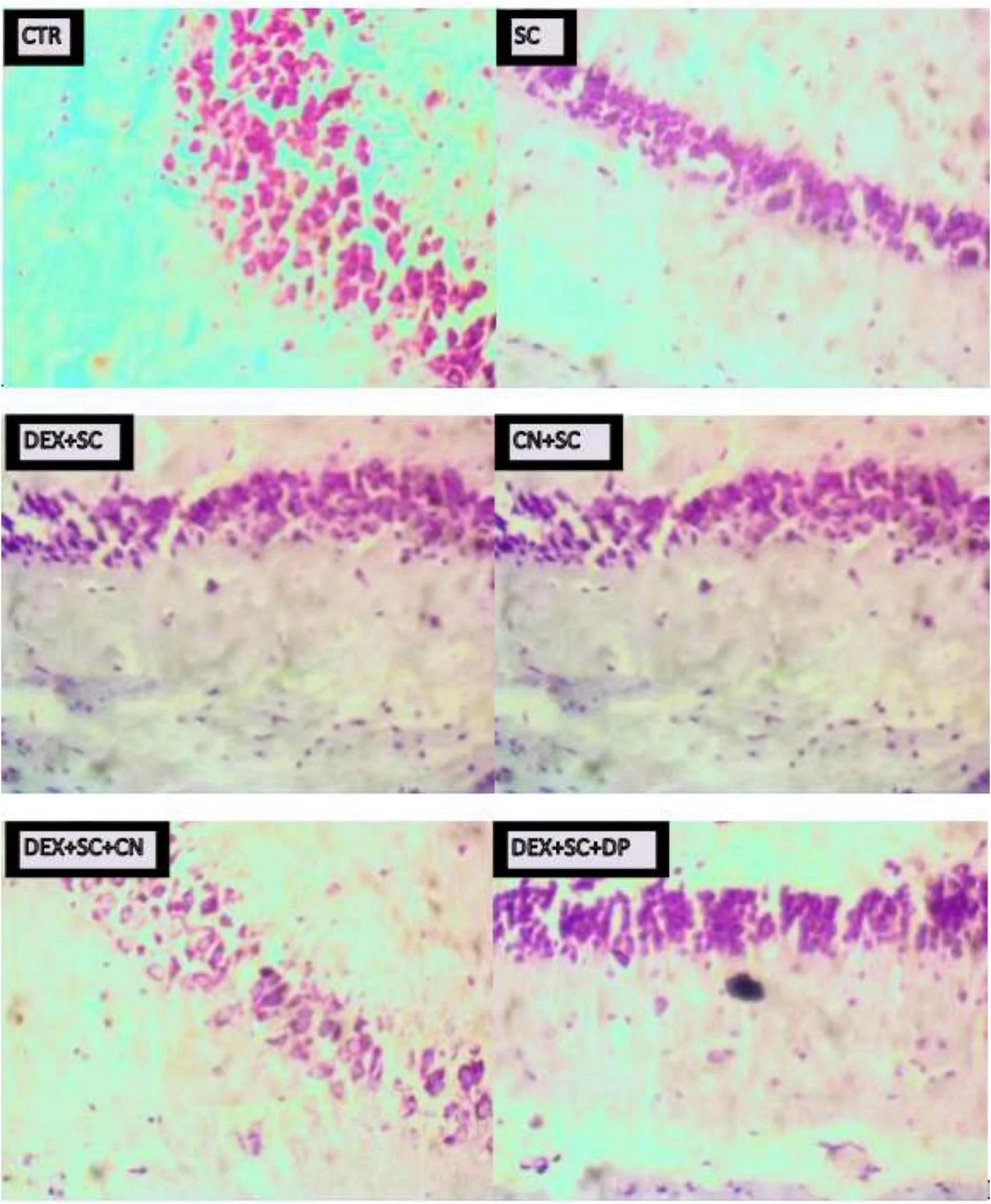
Representative photomicrographs of the effect of methanol extract of *Cola nitida* on the hippocampal area CA1 of mice. The control group has normal morphological organization of the cornu ammonis and normal neuronal cells. SC group shows coagulation necrosis and pyknosis of the pyramidal neurons. Neuronal degeneration, gliosis, and pyknosis were observed in the DEX+SC group. Normal morphological organization of normal neuronal cells are seen in the CN+SC, DEX+SC+CN and DEX+SC+DP groups. Nissl Stain × 400.

Figures 6–8 shows a photomicrograph of the hippocampal regions stained with haematoxylin and eosin, and Nissl. The control group has a normal morphological organization of the cornu ammonis and normal neuronal cells in the CA1, CA2, and CA3 regions. SC group, however, shows coagulation necrosis and pyknosis of the pyramidal neurons in corpus ammonis 1. Likewise, atrophy of the pyramidal neurons was also observed in the cornu ammonis 2 and 3. In the DEX + SC group, coagulation necrosis and pyknosis of the pyramidal neurons were observed in cornu ammonis 1, 2, and 3. The hippocampus with normal morphological organization of the cornu ammonis and normal neuronal cells is seen in CA1, CA2, and CA3 of CN + SC group. In the DEX + SC group, neuronal degeneration, gliosis, and pyknosis were observed in the CA1 and CA3. However, normal morphological organization of the cornu ammonis and normal neuronal cells are seen in CA1, CA2, and CA3 of the DEX + SC + DP group.

### 3.4 Effect of methanol extract of *Cola nitida* on brain acetylcholinesterase

**FIGURE 9:**
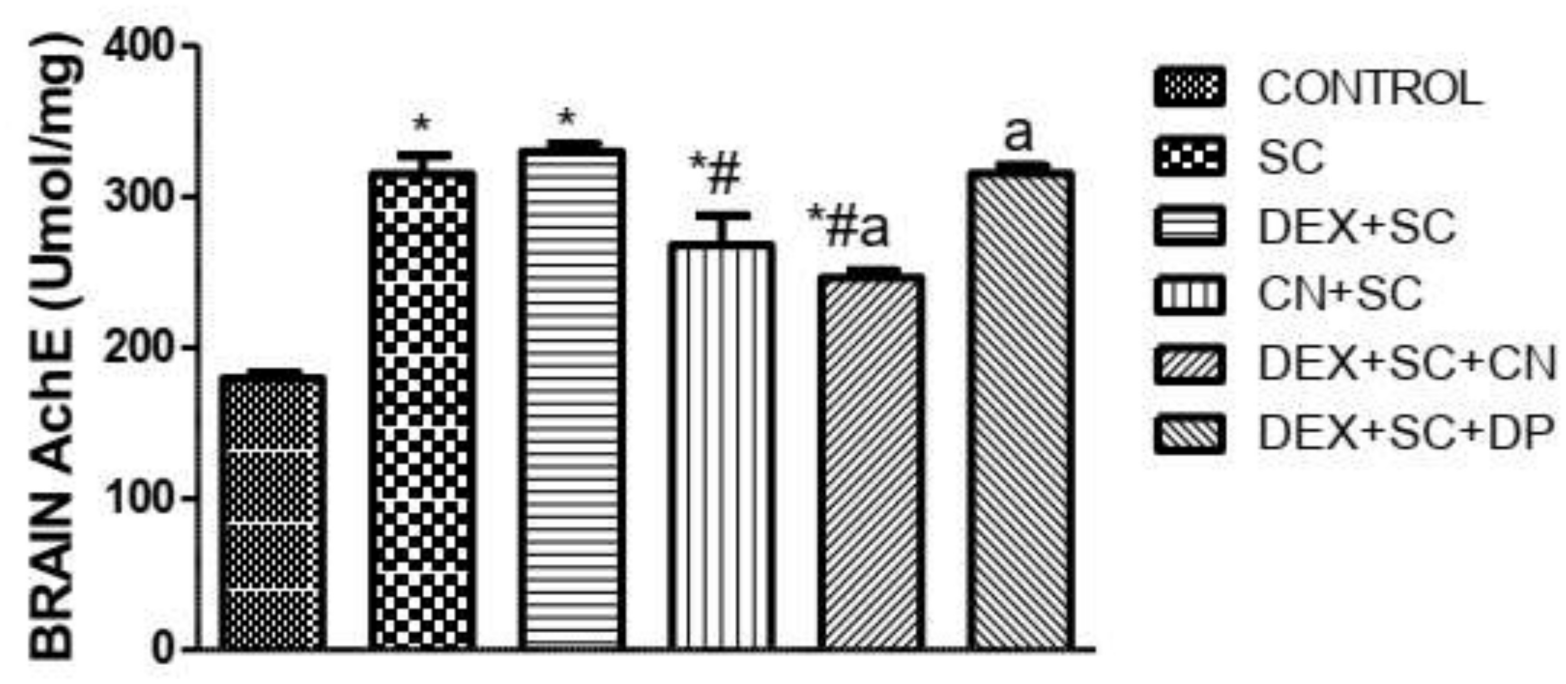
Effect of methanol extract of *Cola nitida* on brain acetylcholinesterase (AchE). *Significant difference from control group at *p* < 0.05. ^#^Significant difference from SC group at *p* < 0.05. ^a^Significant difference from the DEX+SC group. The results are expressed as means ± SEM (*n* = 5). Data were analyzed using One-way ANOVA followed by Newman-Keuls’ *post-hoc* test.

The result in Figure 9 shows that there was a significant increase (*p* < 0.05) in SC, DEX + SC, CN + SC, and DEX + SC + CN treated mice when compared with control group. Also, there was a significant difference (*p* < 0.05) when CN + SC treated group and the DEX + SC + CN treated group were compared with SC treated group. There was a significant decrease (*p* < 0.05) in AchE in DEX + SC + CN and DEX + SC + DP groups when compared with the DEX + SC group.

### 3.5 Effect of methanol extract of *Cola nitida* on lactate and lactate dehydrogenase in the brain and heart

**FIGURE 10:**
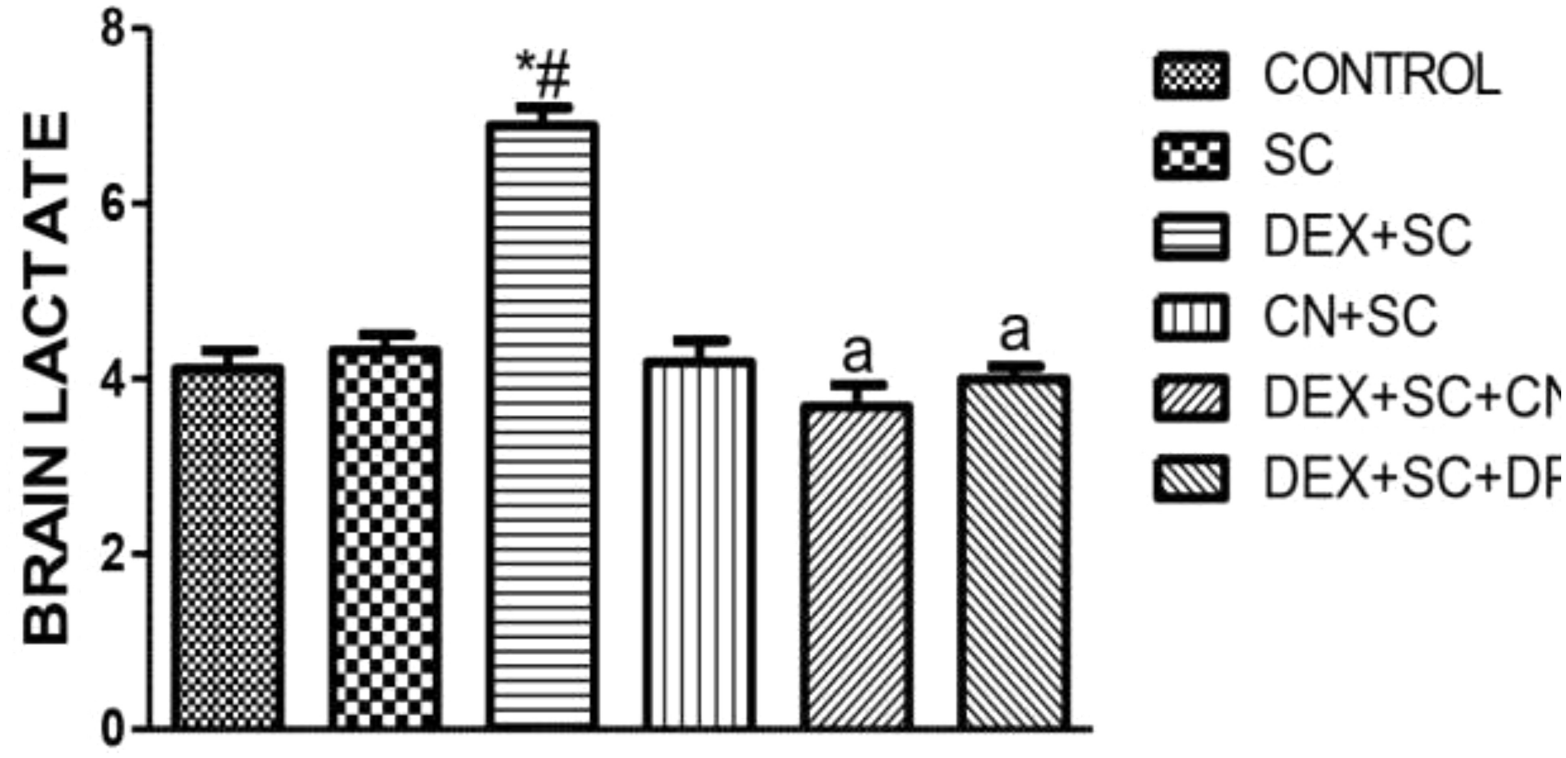
Effect of methanol extract of *C. nitida* on brain lactate. *Significant difference from control group at *p* < 0.05. #Significant difference from SC group at *p* < 0.05. ^a^Significant difference from the DEX+SC group. The results are expressed as means ± SEM (*n* = 5). Data were analyzed using One-way ANOVA followed by Newman-Keuls’ *post-hoc* test.

**FIGURE 11:**
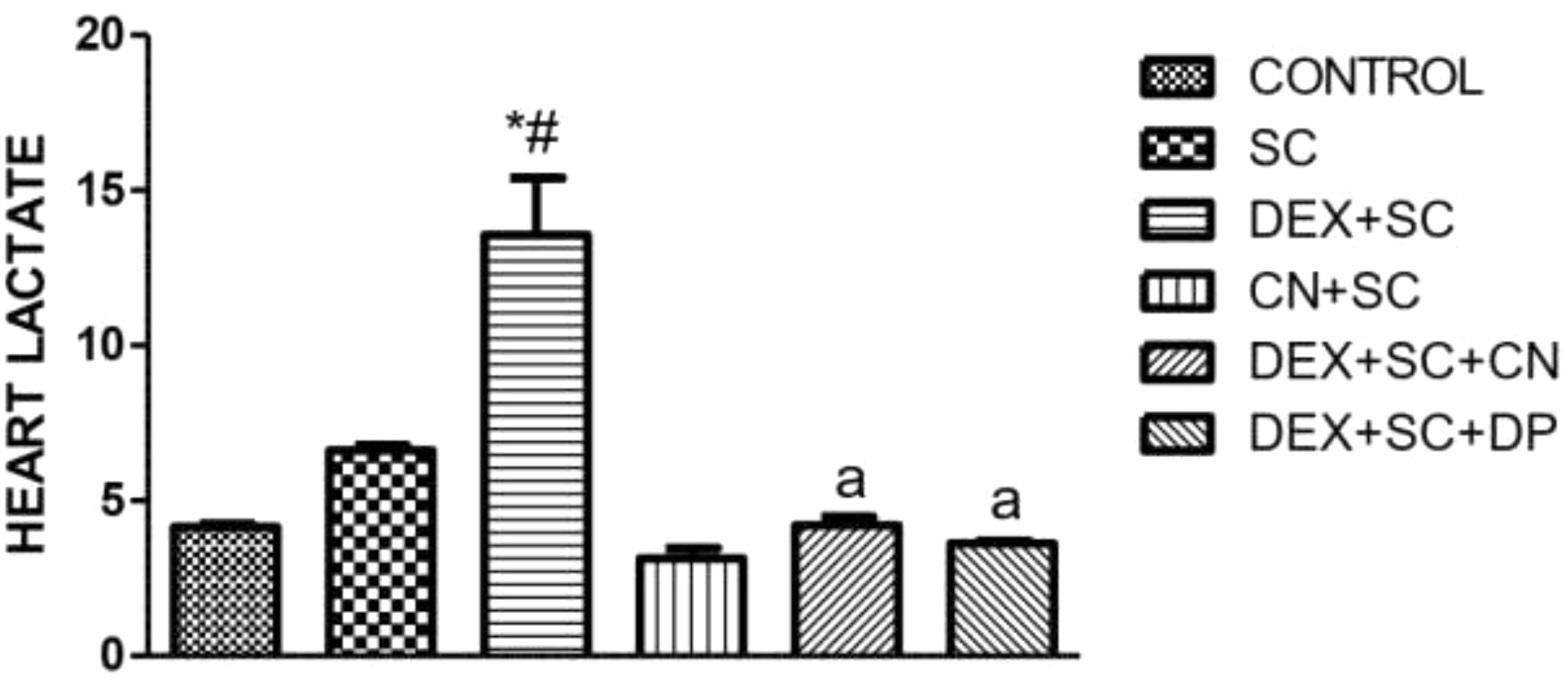
Effect of methanol extract of *C. nitida* on heart lactate. *Significant difference from control group at *p* < 0.05. ^#^Significant difference from SC group at *p* < 0.05. ^a^Significant difference from the DEX+SC group. The results are expressed as means ± SEM (*n* = 5). Data were analyzed using One-way ANOVA followed by Newman-Keuls’ *post-hoc* test.

**FIGURE 12:**
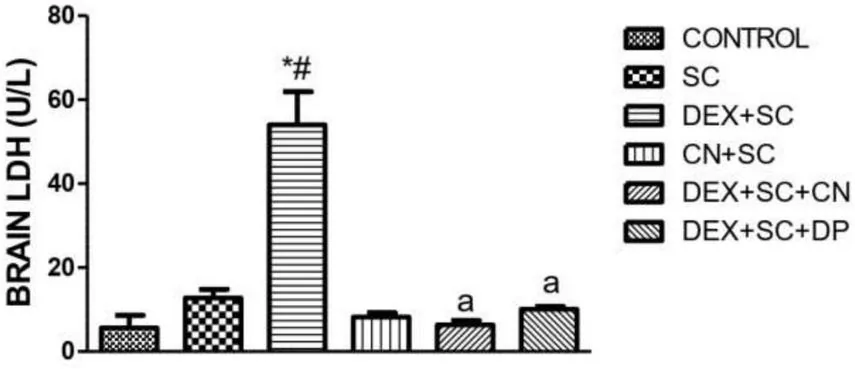
Effect of methanolic extract of *C. nitida* on brain lactate dehydrogenase. *Significant difference from control group at *p* < 0.05. ^#^Significant difference from SC group at *p* < 0.05. ^a^Significant difference from the DEX+SC group. The results are expressed as means ± SEM (*n* = 5). Data were analyzed using One-way ANOVA followed by Newman-Keuls’ *post-hoc* test.

**FIGURE 13:**
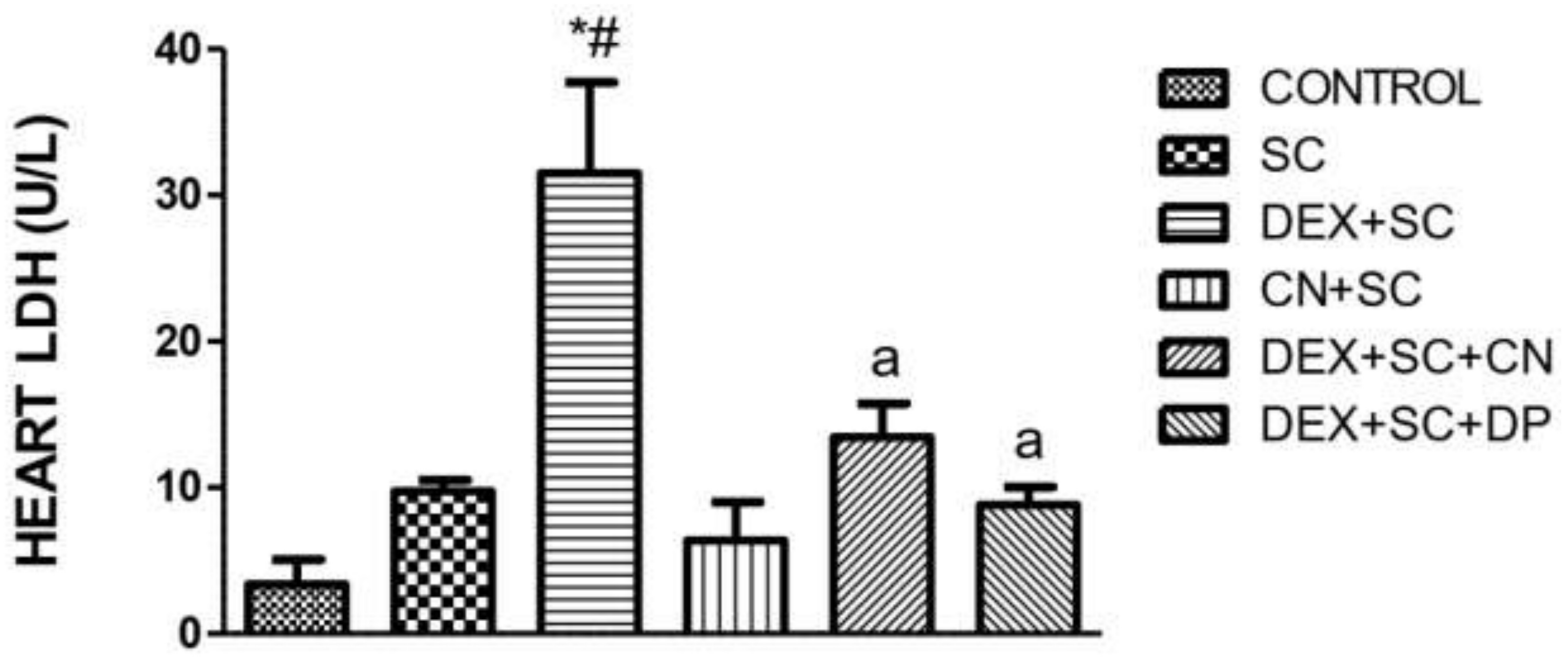
Effect of methanol extract of *C. nitida* on heart lactate dehydrogenase. * = significant difference from control group at *p* < 0.05. ^#^Significant difference from SC group at *p* < 0.05. ^a^Significant difference from the DEX+SC group. The results are expressed as means ± SEM (*n* = 5). Data were analyzed using One-way ANOVA followed by Newman-Keuls’ *post-hoc* test.

Figure 10 shows significant increase (*p* < 0.05) in brain lactate in the DEX+SC group compared to the control and SC groups. The increase was reversed by administration of *C. nitida* and DP is in the significant decrease (*p* < 0.05) in brain lactate in DEX + SC + CN and DEX + SC + CN groups compared to DEX + SC group. Similar results were observed in the heart lactate, brain lactate dehydrogenase, and heart lactate dehydrogenase as shown in Figures 11–13.

### 3.6 Effect of methanol extract of *C. nitida* on glutathione reductase (GR) and reduced glutathione (GSH) in the brain and heart

**FIGURE 14:**
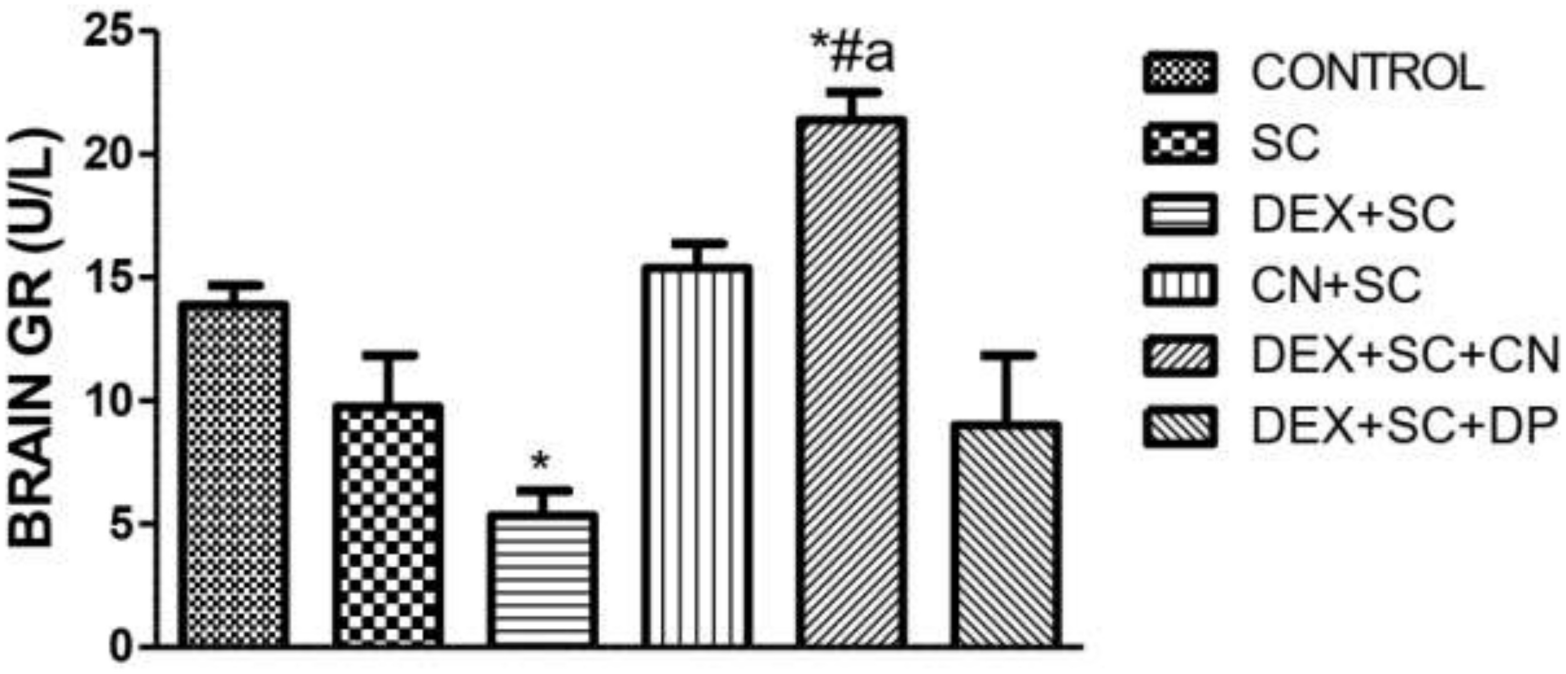
Effect of Methanol extract of *C. nitida* on brain glutathione reductase. *Significant difference from control group at *p* < 0.05. #Significant difference from SC group at *p* < 0.05. ^a^Significant difference from the DEX+SC group. The results are expressed as means ± SEM (*n* = 5). Data were analyzed using One-way ANOVA followed by Newman-Keuls’ *post-hoc* test.

**FIGURE 15:**
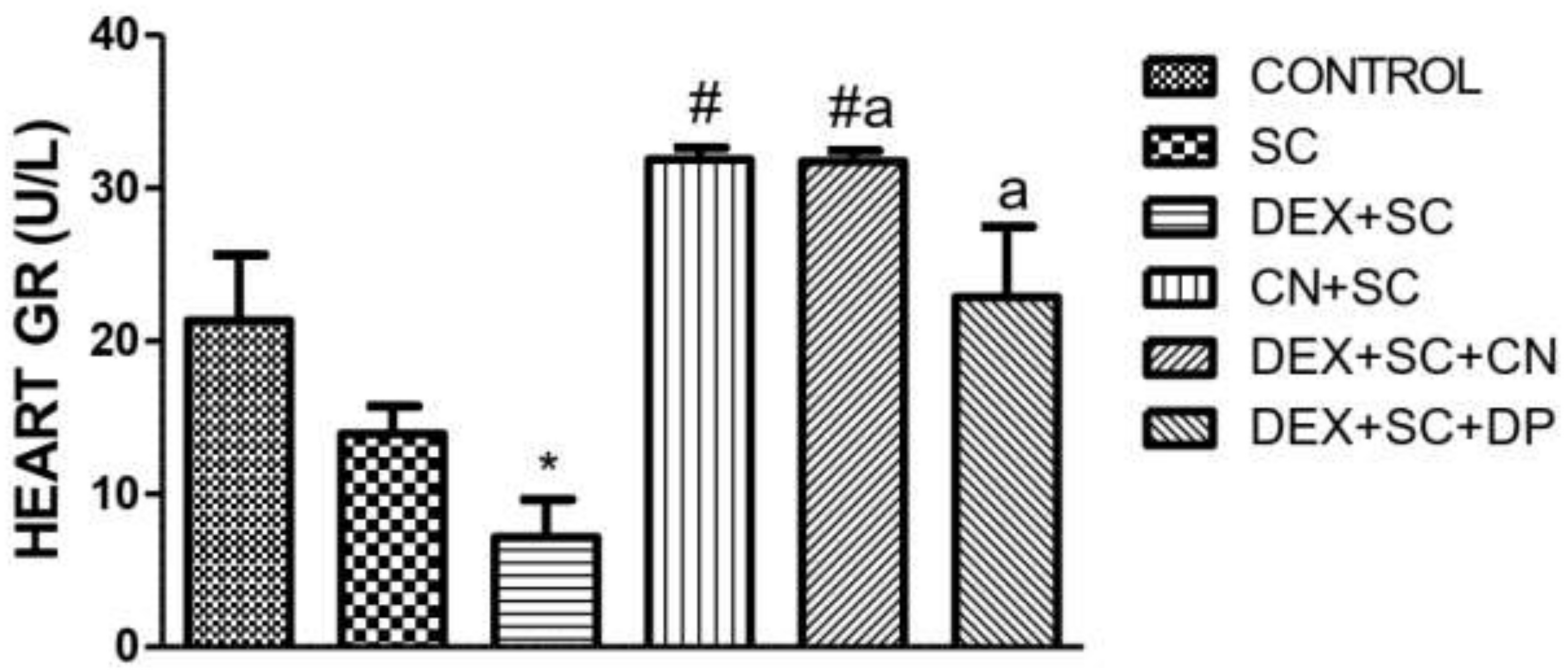
Effect of Methanol extract of *C. nitida* on heart glutathione reductase. * = significant difference from control group at *p* < 0.05. ^#^Significant difference from SC group at *p* < 0.05. ^a^Significant difference from the DEX+SC group. The results are expressed as means ± SEM (*n* = 5). Data were analyzed using One-way ANOVA followed by Newman-Keuls’ *post-hoc* test.

**FIGURE 16:**
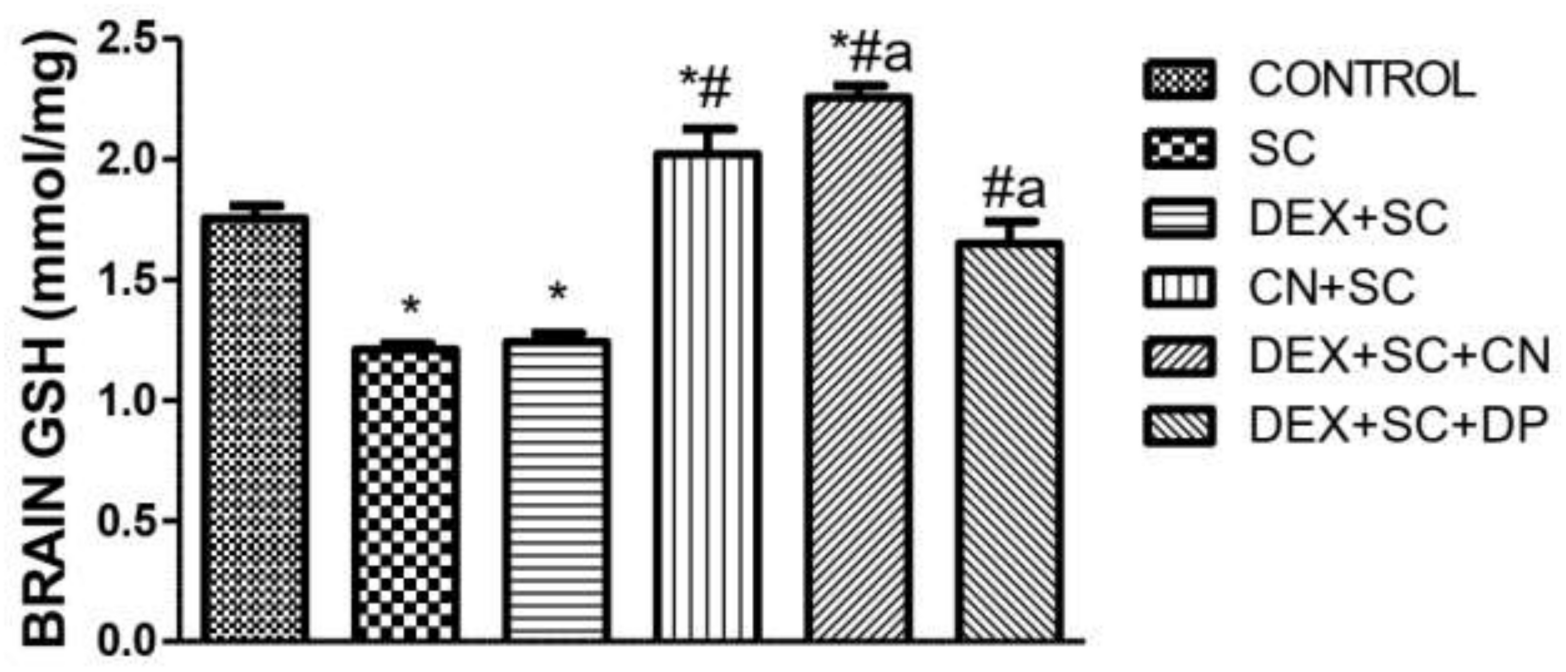
Effect of methanol extract of *C. nitida* on brain GSH. *Significant difference from control group at *p* < 0.05. #Significant difference from SC group at *p* < 0.05. ^a^Significant difference from the DEX+SC group. The results are expressed as means ± SEM (*n* = 5). Data were analyzed using One-way ANOVA followed by Newman-Keuls’ *post-hoc* test.

**FIGURE 17:**
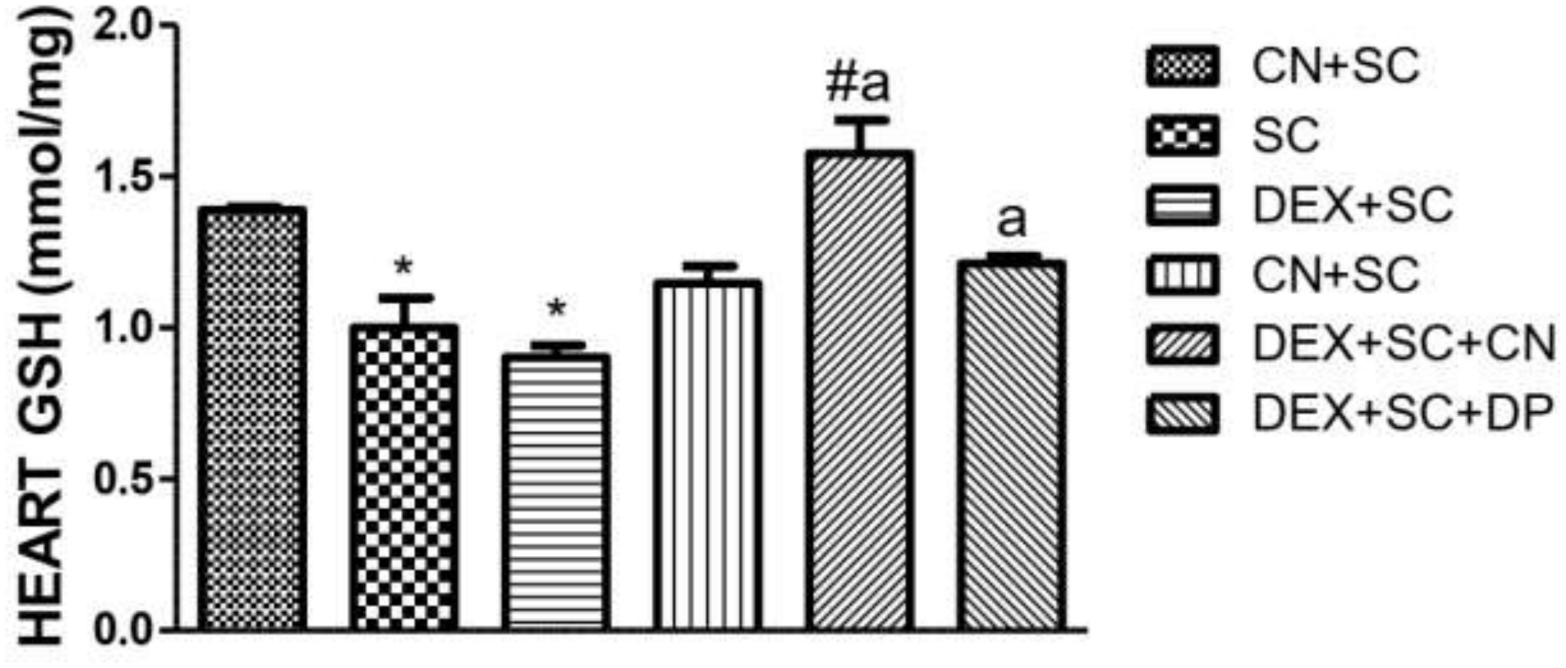
Effect of methanol extract of *C. nitida* on heart GSH. *Significant difference from control group at *p* < 0.05. #Significant difference from SC group at *p* < 0.05. ^a^Significant difference from the DEX+SC group. The results are expressed as means ± SEM (*n* = 5). Data were analyzed using One-way ANOVA followed by Newman-Keuls’ *post-hoc* test.

Figures 14–17 revealed the effect of methanol extract of *C. nitida* on glutathione reductase and GSH in the brain and heart. There was a significant decrease (*p* < 0.05) in glutathione reductase in the brain and heart of mice in the DEX + SC group compared to the control group (Figures 14, 15). There was a significant increase (*p* < 0.05) in glutathione reductase in the brain and heart of the mice in DEX + SC + CN group compared to the DEX+SC group (Figures 14, 15). Furthermore, administration of donepezil significantly increases (*p* < 0.05) glutathione reductase in the DEX + SC + DP group compared to the DEX + SC group.

Similarly, there was a significant reduction (*p* < 0.05) in the reduced glutathione in brain and heart of SC and DEX + SC groups compared to the control group (Figures 16, 17). A significant increase (*p* < 0.05) in GSH was observed in the brain and heart of DEX + SC + CN and DEX + SC + DP groups compared with the DEX + SC groups (Figures 16, 17). Likewise, a significant increase (*p* < 0.05) in GSH was observed in the brain of the CN + SC group compared with the SC group (Figure 4).

### 3.7 Effects of methanol extract of *C. nitida* on MDA and uric acid in the brain and heart

**FIGURE 18:**
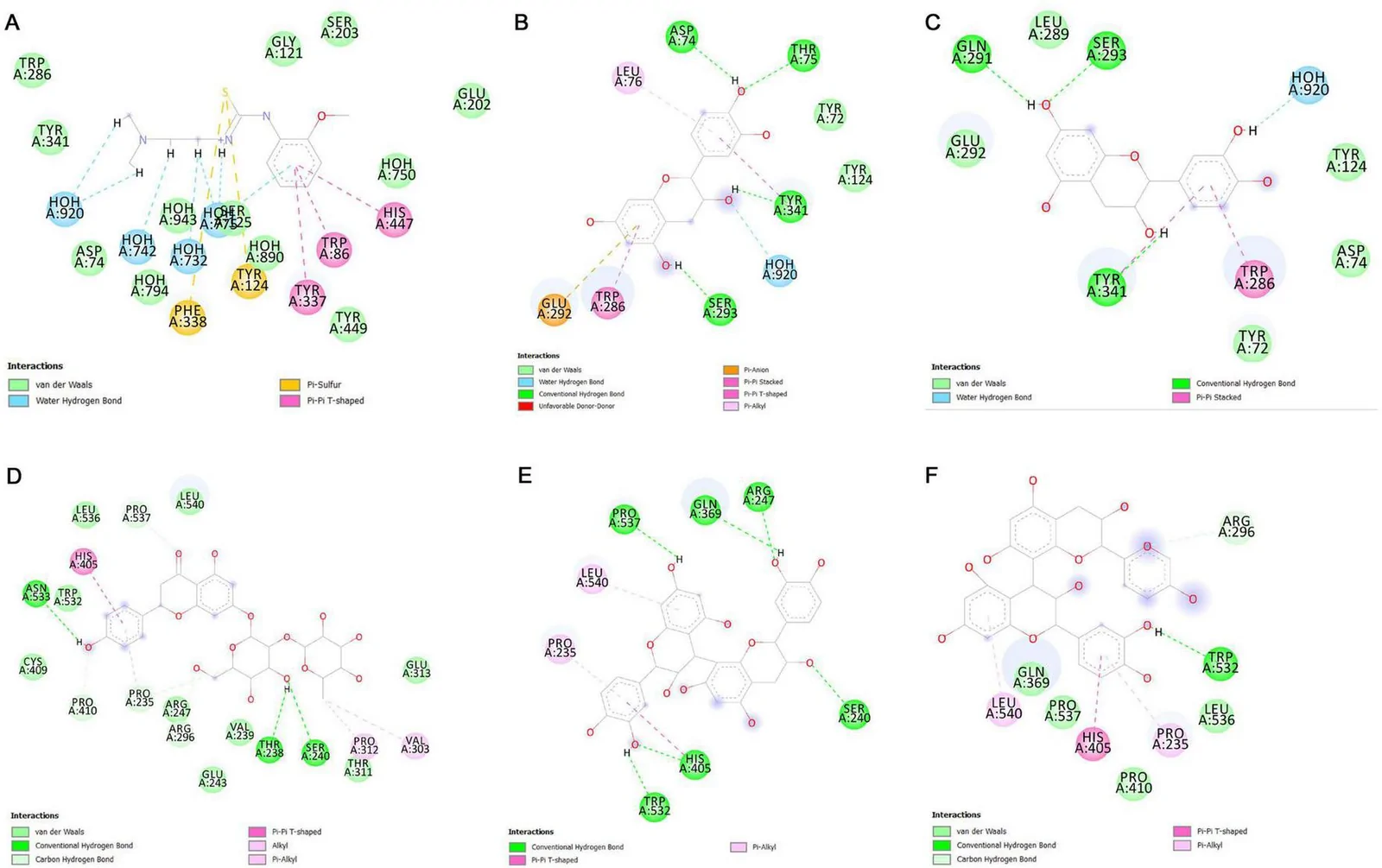
Molecular docking results of flavonoids and procyanidin with the binding site of AchE (A-F). Binding of co-cristallized inhibitor **(A)**, catechin **(B)**, epicatechin **(C)**, naringin **(D)**, procyanidin B1 **(E)** and procyanidin B2 **(F)** with the binding site.

**FIGURE 19:**
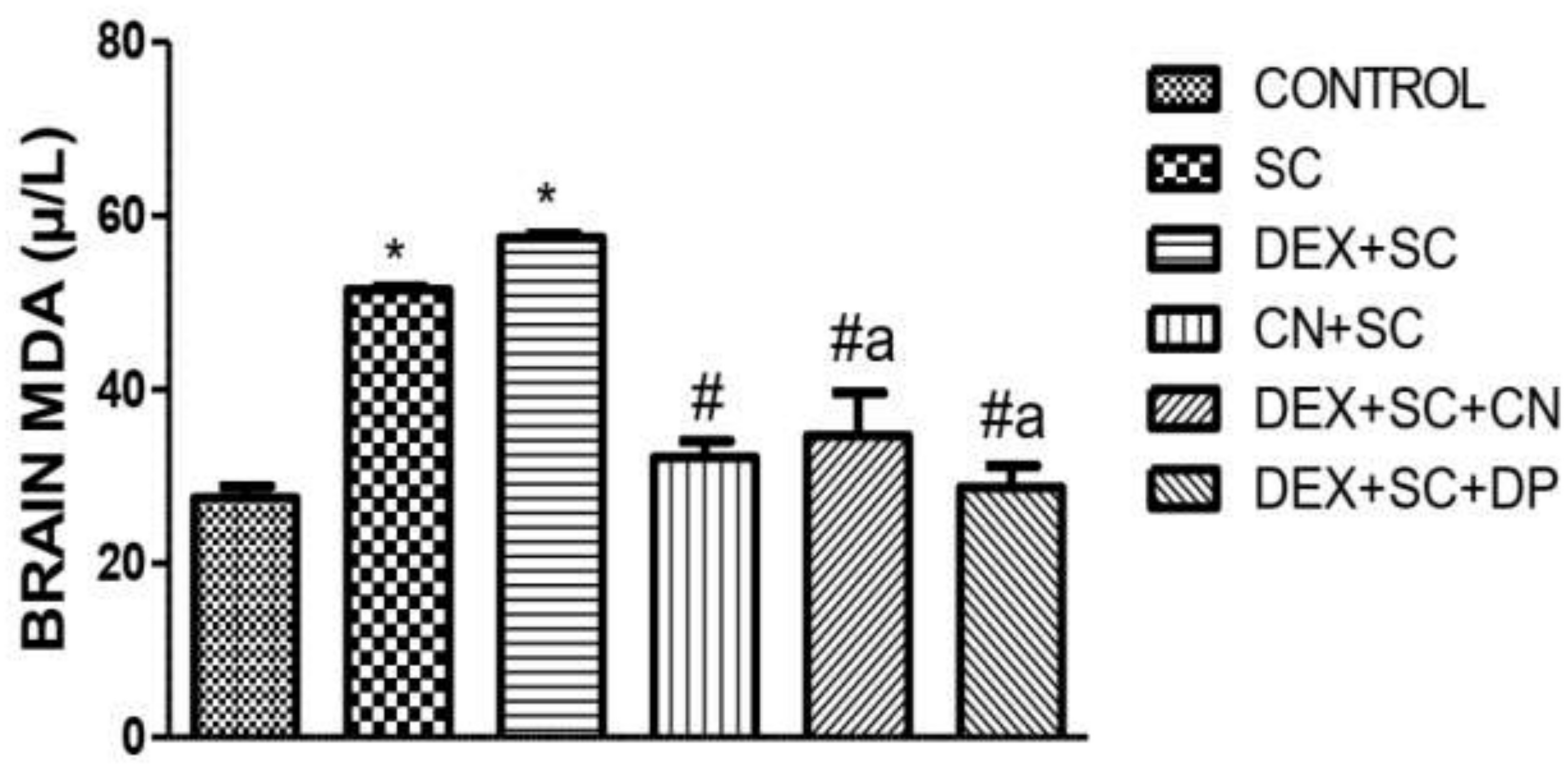
Effect of methanol extract of C. nitida on the brain MDA. *Significant difference from control group at *p* < 0.05. ^#^Significant difference from SC group at *p* < 0.05. ^a^Significant difference from the DEX+SC group. The results are expressed as means ± SEM (*n* = 5). Data were analyzed using One-way ANOVA followed by Newman-Keuls’ *post-hoc* test.

**FIGURE 20:**
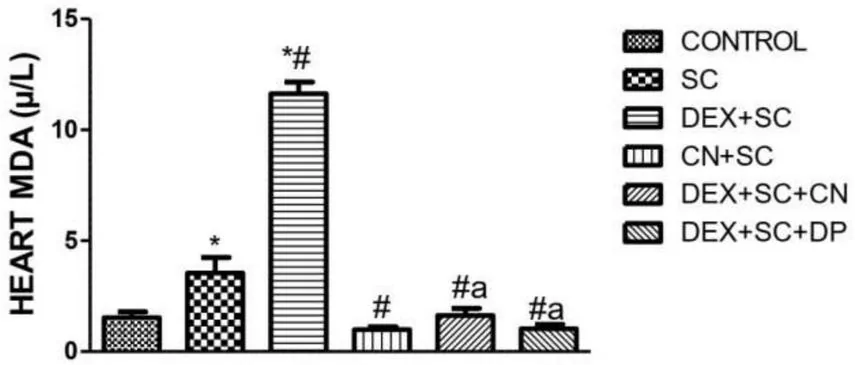
Effect of methanol extract of *C. nitida* on the heart MDA. *Significant difference from control group at *p* < 0.05. ^#^Significant difference from SC group at *p* < 0.05. ^a^Significant difference from the DEX+SC group. The results are expressed as means ± SEM (*n* = 5). Data were analyzed using One-way ANOVA followed by Newman-Keuls’ *post-hoc* test.

Figure 18 shows the effects of the methanol extract of *C. nitida on* MDA and uric acid in the brain and heart. SC and DEX + SC groups had significantly increased (*p* < 0.05) MDA in their brains and hearts compared to the control group (Figures 19, 20). Administration of *C. nitida* significantly reversed (*p* < 0.05) the MDA increase in the CN + SC, DEX + SC + CN, and DEX + SC + DP groups compared to the SC and DEX + SC groups.

**FIGURE 21:**
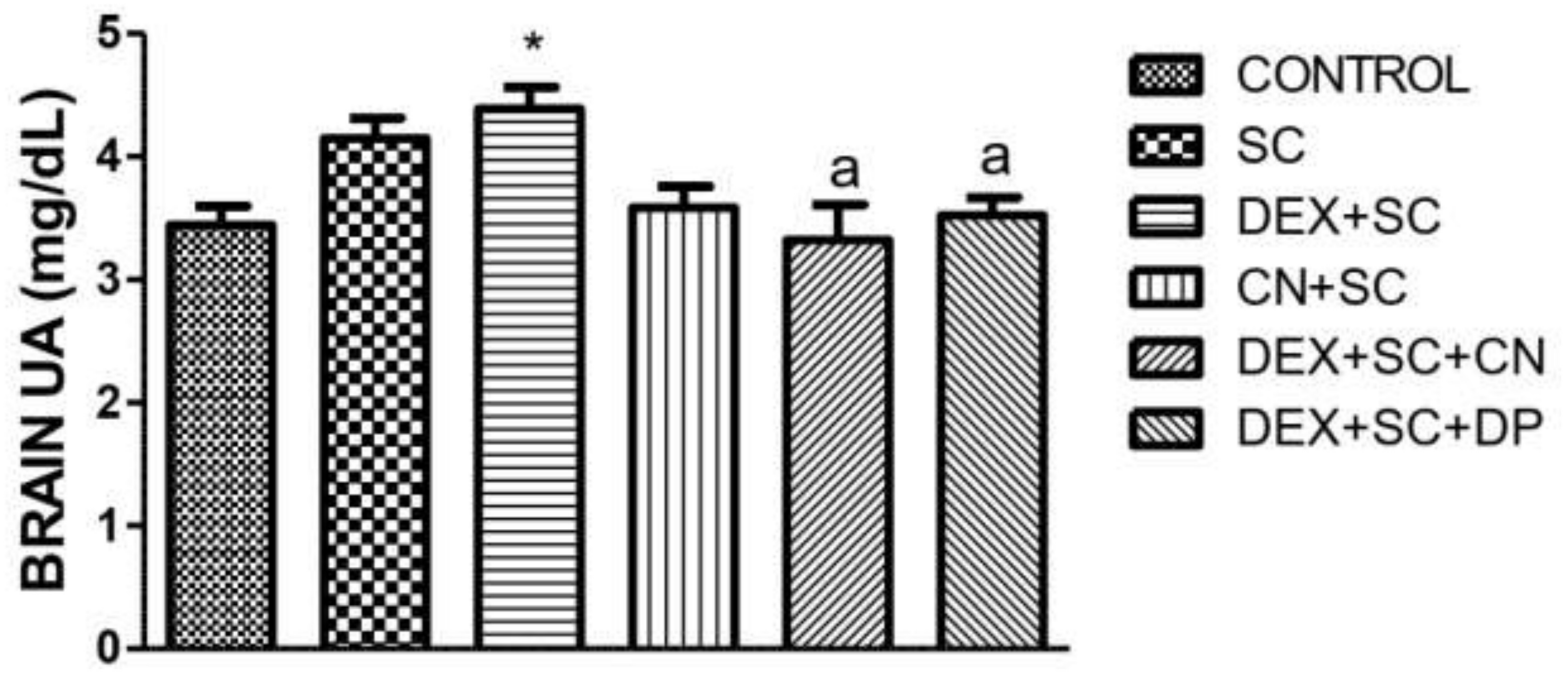
Effect of methanol extract of *C. nitida* on the brain uric acid. *Significant difference from control group at *p* < 0.05. ^a^Significant difference from the DEX+SC group. The results are expressed as means ± SEM (*n* = 5). Data were analyzed using One-way ANOVA followed by Newman-Keuls’ *post-hoc* test.

**FIGURE 22:**
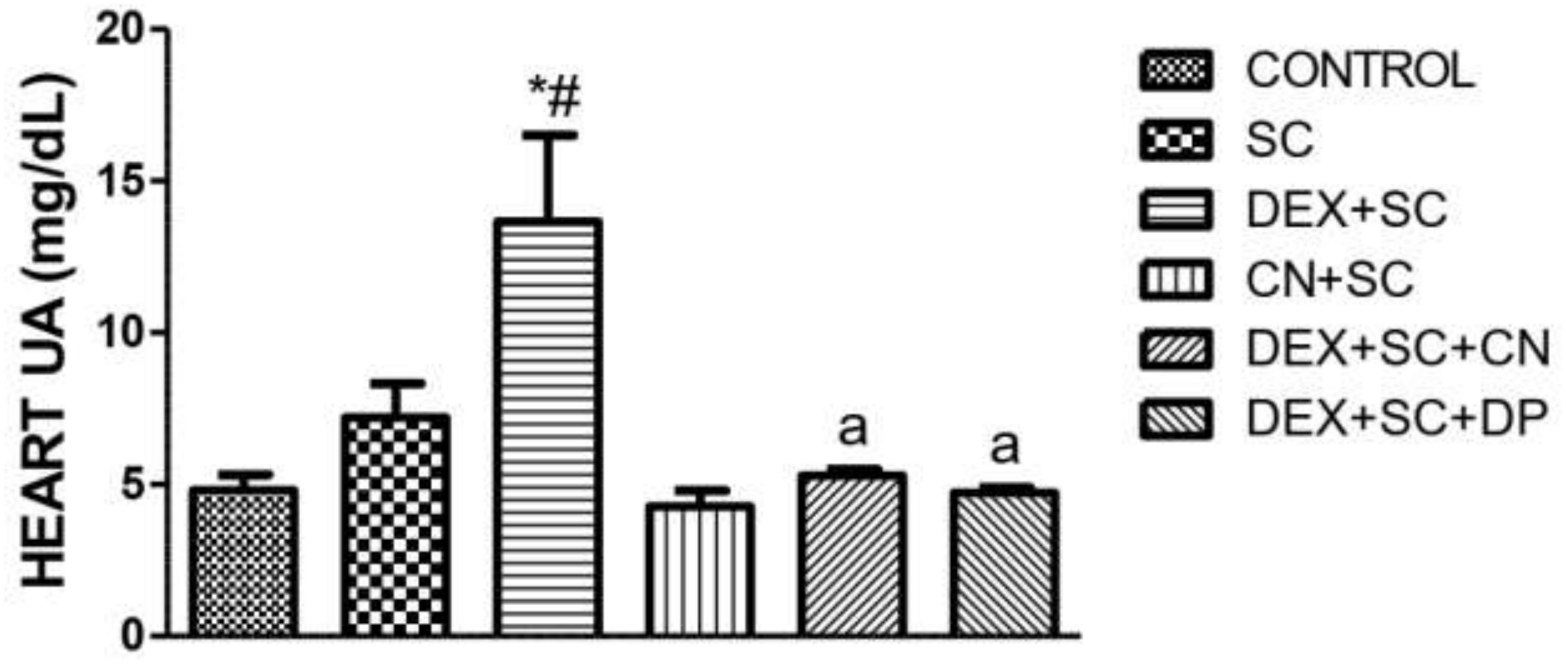
Effect of methanol extract of *C. nitida* on the heart uric acid. *Significant difference from control group at *p* < 0.05. ^#^Significant difference from SC group at *p* < 0.05. ^a^Significant difference from the DEX+SC group. The results are expressed as means ± SEM (*n* = 5). Data were analyzed using One-way ANOVA followed by Newman-Keuls’ *post-hoc* test.

There was a significant increase (*p* < 0.05) in the uric acid in the brain and heart of DEX+SC group compared to the control group (Figures 21, 22). Uric acid was significantly reduced (*p* < 0.05) in the brains and hearts of DEX + SC + CN and DEX + SC + DP groups compared to the DEX + SC group.

### 3.8 Effect of methanol extract of *C. nitida* on triglyceride, high density lipoprotein, total cholesterol, glucose-6-phosphate dehydrogenase (G6PDH) and nitric oxide levels in the brain and the heart

Table 5 shows the effect of the methanol extract of *Cola nitida* on triglyceride, high-density lipoprotein, cholesterol, G6PDH, and nitric oxide levels in the brains and hearts of the mice. Co-administration of DEX and SC significantly increases (*p* < 0.05) the triglyceride level in the heart compared to the control group. Neither *C. nitida* nor DP was able to reverse the triglyceride elevation. There were no significant differences in the HDL, TC, and G6PDH levels across the groups. There was a significant decrease (*p* < 0.05) in the heart nitric oxide in the SC and DEX+SC compared with the control group. Neither *C. nitida* nor DP reversed the nitric oxide decrease in the DEX + SC + CN and DEX + SC + DP groups.

**TABLE 5:** Effect of methanol extract of *C. nitida* on triglyceride, high density lipoprotein, total cholesterol (TC), G6PDH, and nitric oxide levels in the brain and the heart.

Group	CTR	SC	DEX + SC	CN + SC	DEX + SC + CN	DEX + SC + DP
Brain TG (mmol/L)	76.7 ± 0.4	80.6 ± 1.6	75.7 ± 1.17	80.5 ± 0.4	78.5 ± 0.4	81.3 ± 0.4
Heart TG (mmol/L)	85.9 ± 1.3	83.3 ± 2.3	101.9 ± 0.85*	93.2 ± 0.6*	105.6 ± 2.1*	98.2 ± 5.3*
Brain HDL(mmol/L)	11.7 ± 1.1 0	10.3 ± 0.4	8.4 ± 0.3	17.2 ± 5.3	10.5 ± 0.2	13.5 ± 1.3
Heart HDL(mmol/L)	18.8 ± 2.9	26.5 ± 5.1	14.1 ± 2.0	12.9 ± 1.9	10.5 ± 0.2	11.1 ± 1.5
Brain TC (mmol/L)	121.2 ± 1. 7	115.1 ± 1.5	122.8 ± 4.6	112.5 ± 3.1	143.6 ± 9.1	123.7 ± 2.5
Heart TC (mmol/L)	119.5 ± 3. 9	147.1 ± 15.4	125.8 ± 4.7	128.4 ± 6.2	133.6 ± 0.8	132.8 ± 9.9
Brain G6PDH (nmol/mg)	53.3 ± 3.6	50.3 ± 3.8	47.6 ± 0.9	44.1 ± 0.9	51.2 ± 6.2	54.0 ± 2.3
Heart G6PDH (nmol/mg)	54.9 ± 2.7	55.6 ± 0.8	54.7 ± 9.9	54.7 ± 2.0	56.8 ± 0.6	55.1 ± 0.0
Brain NO(m/L)	2.5 ± 0.5	2.6 ± 0.6	3.4 ± 1.3	3.0 ± 0.2	2.5 ± 0.6	3.2 ± 1.1
Heart NO (m/L)	14.2 ± 1.8	7.6 ± 1.0*	5.8 ± 0.4*	3.2 ± 0.1*	9.8 ± 6.6	4.4 ± 1.1*

The results are expressed as means ± SEM (*n* = 5). Data were analyzed using One-way ANOVA followed by Newman-Keuls’ *post-hoc* test. *p < 0.05 compared with control.

### 3.9 Molecular docking results

Molecular docking was performed to evaluate the binding affinities of bioactive components derived from *Cola nitida* with five selected target proteins implicated in glucose metabolism, AChE activity, oxidative stress and memory impairment. The results, summarized in Table 6, indicate that several *Cola nitida*-derived compounds exhibited favorable binding energies, in some cases comparable to or even stronger than those of the co-crystallized ligands. This suggests that these compounds may possess the potential to interact with the selected targets and provide a possible molecular basis for their observed biological effects. However, these docking based predictions should be considered as preliminary evidence and require further experimental validation to confirm target engagement and establish their functional relevance.

**TABLE 6:** Molecular docking results.

Ligand/Protein ID	Co-crystallized ligand	Caffeine	Catechin	Epicatechin	Naringin	Procyanidin B1	Procyanidin B2	Theobromine
3WO3	−10.0	−6.3	−8.6	−8.3	−8.1	−6.7	−7.6	−6.2
6XOX	−15.7	−5.6	−8.0	−7.8	−8.8	−8.1	−8.5	−5.5
8ORC	−6.5	−5.9	−7.9	−8.1	−9.3	−10.0	−10.1	−6.1
7OFE	−9.3	−7.3	−10.5	−10.7	−10.1	−10.2	−10.3	−5.7
4E20	−10.1	−5.7	−9.0	−8.8	−10.2	−8.2	−8.6	−5.7

Regarding glucose metabolism, the tested flavonoids and procyanidins exhibited strong binding affinities toward Akt and GLP-1R, all exceeding −7.5 kcaL/moL. These results suggest that these components may play a key role in modulating glucose-related signaling pathways. Our results also revealed that AchE activity may be potentially inhibited by flavonoids and, interestingly, by procyanidin B1 and B2 that showed the highest affinity for AChE (−10.0 and −10.1 kcaL/moL), significantly surpassing the reference ligand (−6.5 kcaL/moL). Both catechin and epicatechin exhibited stronger interactions with AChE compared to the co-crystallized ligand, recognized as a weak inhibitor. Notably, both compounds interacted with key residues associated with potent AChE inhibition, such as TYR341, TRP286, and GLU292. Additionally, the interaction with the water molecule observed in proximity to TYR124 potentially contributes to the stabilization of the binding.

**FIGURE 23:**
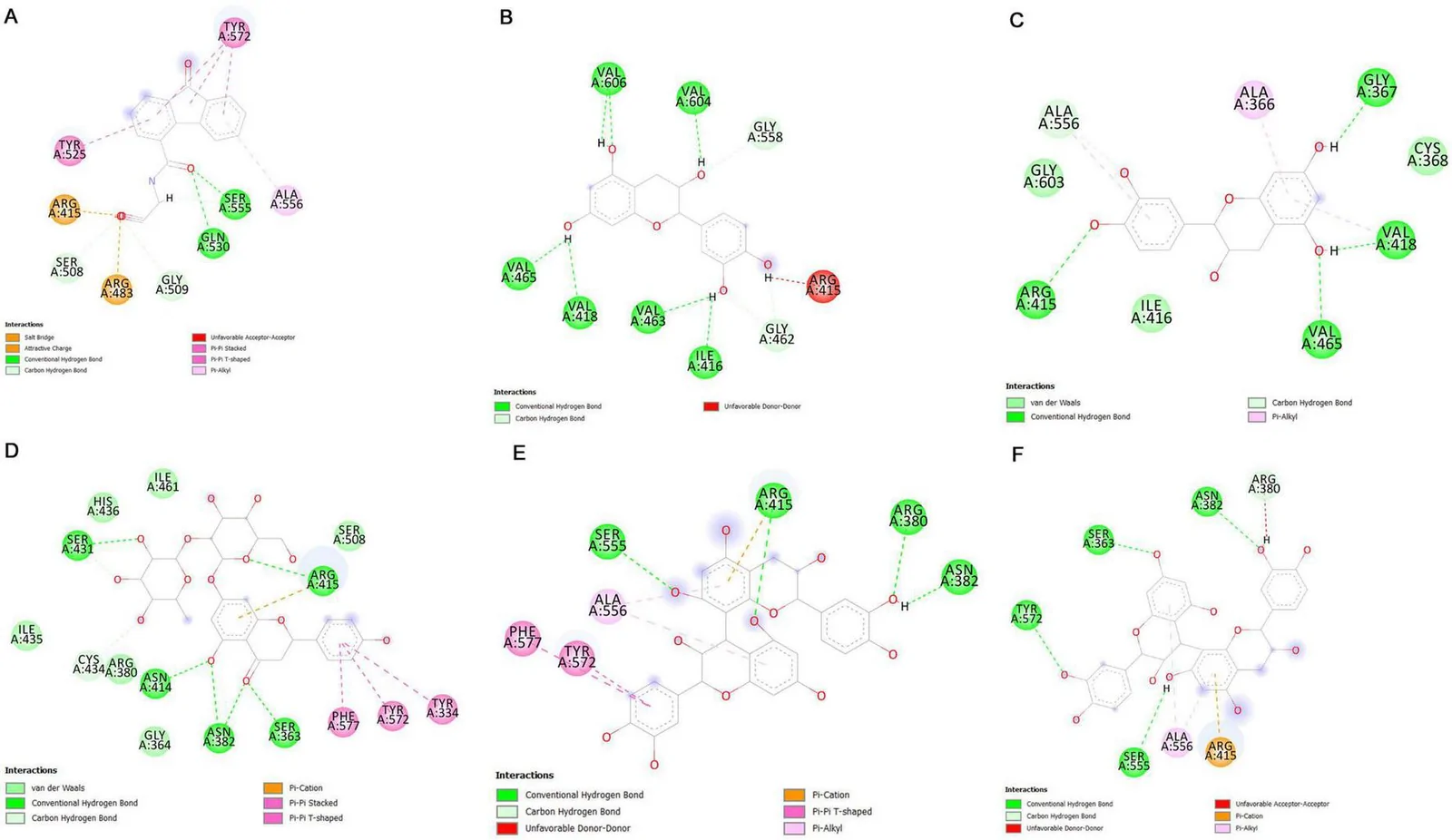
Molecular docking results of flavonoids and procyanidin with the Keap1-Nrf2 recognition site (1-F). Binding pose of the co-cristallized inhibitor **(A)**, catechin **(B)**, epicatechin **(C)**, naringin **(D)**, procyanidin B1 **(E)** and procyanidin B2 **(F).**

Naringin, procyanidin B1, and procyanidin B2 were docked into the same inhibitor binding pocket, but exhibited distinct interaction patterns with key residues within another allosteric site known as site 2 of AChE (Figure 23). Naringin forms three hydrogen bonds with THR238, SER240, and ASN533, along with alkyl and π-alkyl interactions involving residues PRO235, VAL303, and PRO312. Additionally, it establishes a π-π T-shaped interaction with HIS405, a residue critical for stabilizing the ligand-protein interaction. Procyanidin B1 forms hydrogen bonds with ARG247, GLN369, SER240, HIS405, TRP532 and PRO537, forms π-alkyl interactions with LEU540 and PRO253, while also maintaining the π-π T-shaped interaction with HIS405 observed for naringin. Procyanidin B2, in turn, engages in hydrogen bonding with TRP532, retains the π-π T-shaped interaction with HIS405, and conserves the π-alkyl interactions with LEU540 and PRO253 as seen with procyanidin B1. Additionally, it forms a carbon-hydrogen bond with ARG296. All these residues are known to define the druggable allosteric site 2 of AChE, highlighting the potential of these components as modulators of AChE activity.

In the context of oxidative stress, most tested compounds with the exception of the alkaloids caffeine and theobromine, demonstrated strong binding affinities ranging from -10.1 to -10.7 kcal/mol, compared to -9.3 kcal/mol for the co-crystallized inhibitor (Table 6). The interaction between the transcription factor nuclear factor erythroid 2 related factor 2 (Nrf2) and its repressor Keap1 is primarily mediated by two motifs in Nrf2: the high-affinity ETGE motif and the low-affinity DLG motif. The Keap1 binding site consists of six subpockets, designated P1 through P6. As shown in Figure 23, catechin occupies the same binding pocket as the co-crystallized inhibitor, forming a carbon-hydrogen bond with GLY462 and establishing a total of seven hydrogen bonds. However, it does not interact with the key amino acids typically involved in the Keap1-Nrf2 interaction. In contrast, epicatechin forms four hydrogen bonds, notably with ARG415 located in the P1 subpocket. It also engages in hydrophobic interactions, including carbon and π-alkyl contacts contributing to ligand stability, with ALA556- an amino acid known to play a critical role in the Keap1-Nrf2 binding interface.

Naringin interacts with SER431 and CYS434 within the P6 subpocket, which is known to mediate the low-affinity binding of Keap1 to the DLG motif of Nrf2. Additionally, it forms a salt bridge with ARG415 in the P1 subpocket, mimicking the key interaction of the ETGE motif, responsible for high-affinity binding. The compound also establishes hydrogen bonds with ASN414, ASN382, and SER363 within P2, and engages in hydrophobic interactions with TYR334, TYR572, and PHE577 from P5. These multi-subpocket interactions suggest that naringin effectively mimics the native Nrf2 binding pattern, thus disrupting both anchoring mechanisms required for Keap1-mediated ubiquitination.

Procyanidin B1 and B2 bind across key subpockets of the Keap1 Kelch domain P1, P2, P3, and P5, suggesting strong mimicry of the high-affinity ETGE motif of Nrf2. It interacts with TYR 572, TYR577, SER555, and ALA556 in P3 and P5, and forms a critical salt bridge with ARG415 in P1. Additional hydrogen bonds with ARG380 and ASN382 in P2 further reinforce its alignment with the ETGE binding interface. These interactions indicate that procyanidins effectively target the core ETGE-binding site, potentially disrupting Keap1-Nrf2 recognition.

**FIGURE 24:**
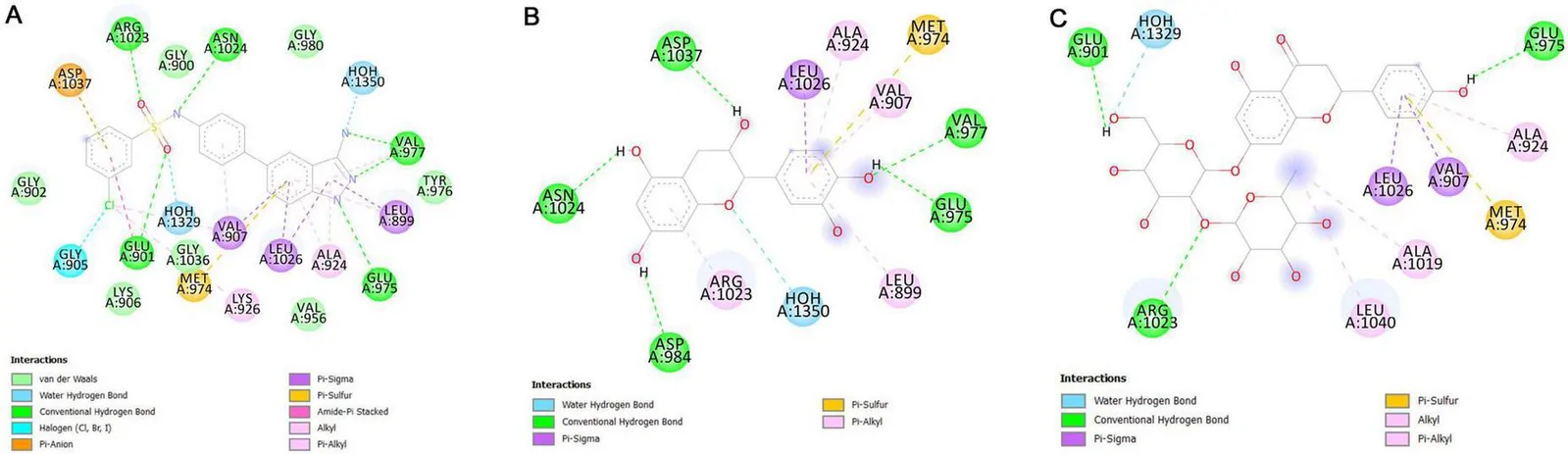
Molecular docking results: Binding of co-crystallized ligand **(A)**, catechin **(B)** and naringin **(C)** with the active site of Tyk2.

As for Tyk2, molecular docking results revealed that flavonoids and procyanidin displayed strong interactions with the binding site, with affinities ranging from -8.2 to -10.2 kcaL/moL, the strongest being observed for the Tyk2–naringin complex (Table 6). Naringin appears to be the most potent modulator of Tyk2 activity, displaying a binding affinity of -10.2 kcaL/moL, slightly stronger than that of the co-crystallized ligand. It engages in similar key interactions, including π-sulfur with MET974, π-alkyl with ALA924, and π-sigma interactions with VAL907 and LEU1026, as well as several hydrogen bonds with GLU901, GLU975, and ARG1023 that contribute to the stability of the complex (Figure 24).

## 4 Discussion

The present study demonstrated that oral administration of *Cola nitida* methanol extract significantly attenuated scopolamine- and dexamethasone-induced cognitive and cardiac metabolic dysfunction in male mice. Specifically, *Cola nitida* effectively reversed scopolamine-induced memory deficits, as evidenced by improved performance in both the Morris Water Maze and Y-Maze tests. Treatment with *Cola nitida* significantly reduced escape latency and increased the time spent in the target quadrant in the Morris Water Maze, while also improving spontaneous alternation in the Y-Maze compared with mice treated with scopolamine or dexamethasone plus scopolamine alone. In addition, *Cola nitida* restored body weight and improved biochemical, functional, histological, and behavioral parameters. Biochemical analyses showed that the extract reduced acetylcholinesterase activity and decreased malondialdehyde and uric acid levels in both the brain and heart. Furthermore, *Cola nitida* normalized lactate and lactate dehydrogenase levels and preserved hippocampal histoarchitecture. Collectively, these findings indicate that *Cola nitida* exerts neuroprotective and cardiometabolic effects, likely through its antioxidant, lipid-regulating, and cholinergic-modulating properties.

Neurological and cardiac dysfunction are known to significantly impair cognitive functions such as learning, spatial memory, working memory, and long-term memory. Hippocampus, an important region of the brain necessary for cognitive functioning, is particularly vulnerable to both metabolic dysfunction and neurodegeneration (Kim et al., 2024). Results from the current study showed neurodegeneration in CA1, CA2 and CA3 of mice that were administered scopolamine, and scopolamine and dexamethasone. The observed neurodegeneration is reflected in the impaired cognitive functions recorded in these groups. The CN-treated group, however, showed normal morphological organization of the cornu ammonis, normal neuronal cells, and no lesion.

One of the proposed mechanisms by which cardiometabolic diseases affect cognitive functioning is insulin resistance. Insulin resistance impairs synaptic plasticity and neurotransmitter activity essential for learning (Spinelli et al., 2019). Glucose intolerance was observed in our study in the DEX+SC group as reflected in a significant increase in the Area Under the Curve (AUC) of the Oral Glucose Tolerance Test in this group compared to the control group. Elevated 1-h postload glucose is a reliable independent determinant of impaired pancreatic β-cell function and insulin resistance that are accompanied by increased atherosclerotic cardiovascular disorder (Bianchi et al., 2013). Our findings revealed that *C. nitida* administration significantly reduced the AUC of the oral glucose tolerance test. Therefore, *C. nitida* preserved cognitive functions in dexamethasone- and scopolamine-induced cardiometabolic and memory dysfunction by protecting the hippocampal neurons from neurodegeneration and possibly enhancing glucose tolerance.

Scopolamine is a muscarinic acetylcholine receptor antagonist that blocks muscarinic receptors in regions like the hippocampus, which are necessary for learning and memory. The blockade of muscarinic receptors leads to a compensatory increase in AChE activity, as seen in SC and DEX+SC groups in our study. Scopolamine thus induces cognitive impairment by disrupting the cholinergic neurotransmission (Lochner and Thompson, 2016). A reduction in AChE activity was observed in *C. nitida-treated* groups, indicating the restoration of cholinergic function. *C. nitida* might have reduced AChE by blocking the enzyme’s activity.

Lactate and lactate dehydrogenase (LDH) levels in the heart and brain can be assessed to evaluate metabolic stress, tissue damage, impaired oxidative phosphorylation, and neurotoxicity (Han et al., 2023). Increased lactate and LDH levels are associated with impaired memory in experimental animals (Zhao et al., 2022). DEX administration significantly increases lactate and LDH levels in the hearts and brains of DEX+SC mice, an effect that was reversed in CN-treated groups. Furthermore, oxidative stress has been implicated as a major mechanism in the development of cardiometabolic dysfunction and neurodegenerative conditions associated with cognitive decline (Franzoni et al., 2021). Likewise, natural antioxidants are proven to reverse cognitive decline in neurodegenerative diseases (Franzoni et al., 2021). In our study, scopolamine and dexamethasone induced oxidative stress by increasing uric acid and MDA levels and depleting glutathione (GSH) in the hearts and brains of the SC and DEX + SC groups. *C. nitida* a known natural antioxidant (Adedayo et al., 2019), ameliorated the oxidative impacts of SC and DEX + SC administration in the treated groups. Glutathione (GSH) and glutathione reductase (GR) are pivotal in maintaining redox balance within the heart and brain. In the cardiovascular system, diminished GSH levels compromise antioxidant defenses, leading to oxidative stress, endothelial dysfunction, and myocardial injury, which can impair cardiac function (Tan et al., 2023). Similarly, in the brain, reduced GSH availability is associated with increased oxidative damage, neuronal loss, and cognitive decline (Chen et al., 2022). Notably, studies have demonstrated that lower plasma GSH levels correlate with cognitive deterioration in individuals with mild cognitive impairment (MCI) over 2 years (Lin and Lane, 2021). Also, cardiac nitric oxide bioavailability was reduced in the SC and DEX+SC groups, indicating endothelial dysfunction, which may be due to elevated uric acid and oxidative stress noticed in these mice. Interestingly, brain nitric oxide, G6PDH, and cardiac G6PDH levels were not altered. These findings underscore the significance of GSH and GR in mitigating oxidative stress and highlight their potential as therapeutic targets in managing cardiac and cognitive disorders.

Elevated uric acid in both circulation and target organs, such as the heart and brain, has been linked to cognitive impairment. Hyperuricemia promotes endothelial dysfunction, oxidative stress, and cardiac remodeling, which consequently impair cerebral perfusion and increase neuroinflammation (Feig et al., 2008; Ndrepepa, 2018). Within the brain, uric acid metabolism via xanthine oxidase generates reactive oxygen species that disrupt neuronal integrity and synaptic signaling (Sautin and Johnson, 2008). Clinical studies further show that higher serum uric acid is associated with accelerated cognitive decline and increased dementia risk, particularly in individuals with cardiometabolic disorders (Khan et al., 2016; Latourte et al., 2018). Hence, our findings highlight uric acid as both a biomarker and mechanistic mediator linking scopolamine and dexamethasone-induced cardiac dysfunction to neurodegeneration.

Elevated levels of malondialdehyde (MDA) in the hippocampus had damaging effects on spatial learning and memory capability by impairing the ultra-microstructure of the hippocampal CA1 area (Alves Oliveira et al., 2020). The group treated with CN (DEX + SC + CN) showed a significant decrease in lipid peroxidation in the brain as indicated by MDA levels when compared to the DEX + SC treated group. This connotes that CN exhibits a therapeutic effect on spatial learning and amnesia by its antioxidative properties and preservation of the hippocampal neurons. In addition, CN-treated groups had significantly reduced heart MDA levels compared to the DEX + SC group, showing that CN could ameliorate cardiac lipid peroxidation by its therapeutic effect on the heart MDA.

In the present study, the elevation in cardiac triglyceride levels induced by dexamethasone and scopolamine was unaltered by treatment with either *Cola nitida* extract or donepezil. Hence, CN and donepezil treatment did not exacerbate cardiac TG accumulation caused by DEX + SC treatment. Therefore, Cola nitida or donepezil did not worsen cardiac lipid dysregulation in mice with cardiac dysfunction and cognitive impairment.

In our model, we observed that dysregulated cardiac triglycerides, increased LDH/lactate, elevated acetylcholinesterase (AChE) activity, and markers of oxidative stress were all associated with impaired spatial and working memory. These findings are consistent with multiple lines of evidence showing that cardiac and brain dyslipidemia contribute to cognitive decline via both systemic and central mechanisms. For example, an elevated LDL/HDL cholesterol ratio in older adults has been linked to worse cognitive performance and increased risk of dementia (Wen et al., 2024). High cholesterol in LDL receptor-deficient mice also impairs spatial memory, reduces mitochondrial complex activity, decreases glutathione, and increases lipid peroxidation in the brain (de Oliveira et al., 2011). Meanwhile, in diabetes-associated cognitive decline, rising lactate and LDH-A expression correlate with memory impairment, and blocking LDH-A can ameliorate these deficits (Zhao et al., 2018). Collectively, these data reinforce that dyslipidemia induces oxidative stress, disrupts energy metabolism (e.g., via LDH/lactate), heightens cholinergic dysfunction (as shown by increased AChE), and ultimately contributes to neurodegeneration and cognitive impairment.

Our molecular docking results revealed that most tested compounds, except for the alkaloids caffeine and theobromine, may modulate target protein activities. The molecular docking results suggest that the flavonoids and procyanidins have strong binding affinities toward Akt and GLP-1R, suggesting a potential role in modulating glucose-related signaling pathways. In addition, AChE activity may be potentially inhibited by flavonoids and, interestingly, by procyanidin B1 and B2. While catechin and epicatechin show strong interactions with key residues associated with potent AChE inhibition (Kumari et al., 2024), naringin, procyanidin B1, and procyanidin B2 exhibited distinct interaction patterns with key residues within another druggable allosteric site known as site 2 of AChE (Roca et al., 2018), highlighting the potential of these components to modulate the AChE activity in different ways.

In the context of oxidative stress, our results demonstrated strong binding affinities with the key amino acids typically involved in the Keap1-Nrf2 interaction (Crisman et al., 2023; Qin et al., 2024). While catechin and epicatechin display the highest binding energies, they do not interact with the well-known residues involved in the Keap1-Nrf2 complex interaction. However, Procyanidin B1 and B2 strongly mimic the binding of the high-affinity ETGE motif of Nrf2, while naringin mimics the native Nrf2 binding pattern, thus disrupting both anchoring mechanisms required for Keap1-mediated ubiquitination. These findings highlight the potential role of *Cola nitida* components as modulators of the Keap1-Nrf2 interaction and reflect their distinct binding thermodynamics and kinetics profile. As for Tyk2, molecular docking results suggests that naringin appears to be the most potent modulator of Tyk2 activity, engaging in similar key interactions as of the co-crystallized ligand.

Furthermore, our molecular docking results give a preliminary mechanistic understanding. Given the stability and potential biologically activeness of the interactions of flavonoid and procyanidin with Akt, GLP-1R, AChE, and Keap1-Nrf2, it is reasonably anticipated that these protein-ligand interactions would continue in dynamic situations, as practically established through molecular dynamic simulations. These computational results, in effect, reinforce the experimental evidence by proposing that CN may exert its mechanism of action on neuro-metabolic processes through multi-target regulations.

Our findings demonstrate behavioral, biochemical, and histological effects of *C. nitida*, while the molecular docking results serve only as hypothesis-generating evidence that identifies potential molecular targets for future mechanistic investigations. We acknowledged that confirmation of these proposed mechanisms will require dedicated molecular studies, including protein and gene expression analyses (e.g., Keap1, Nrf2, TYK2, AKT, GLP-1R) and pathway-specific functional assays. A limitation of the present study is that the methanol extract of *Cola nitida* was not chemically standardized using chromatographic techniques such as HPLC or LC-MS. The docking analyses were therefore based on phytochemicals previously reported in the literature for *Cola nitida.*

## 5 Conclusion

In conclusion, oral administration of 50 mg/kg *Cola nitida* (CN) methanol extract significantly attenuated dexamethasone- and scopolamine-induced cognitive impairment and cardiac metabolic dysfunction in male mice. These protective effects were associated with preservation of hippocampal neuronal integrity, attenuation of oxidative stress, and inhibition of acetylcholinesterase (AChE) activity, collectively contributing to improved cognitive and cardiac function. Molecular docking analyses further demonstrated that bioactive flavonoids and procyanidins present in *Cola nitida* exhibit favorable binding affinities for proteins involved in glucose metabolism, oxidative stress regulation, and cholinergic neurotransmission, providing mechanistic support for the observed biological effects. Overall, these findings identify *Cola nitida* as a promising natural therapeutic candidate for mitigating cardiac metabolic disturbances and cognitive decline. Nevertheless, additional preclinical and clinical studies are needed to establish its long-term safety, efficacy, pharmacokinetic profile, and translational potential in humans.

Our future study will explore the molecular mechanisms underlying CN’s effects, particularly its influence on insulin signaling pathways and cholinergic neurotransmission. Furthermore, dose-response relationships will be explored in future studies with extended treatment duration.

## Data Availability

The original contributions presented in the study are included in the article/supplementary material, further inquiries can be directed to the corresponding authors.

## Footnotes

1
https://pubchem.ncbi.nlm.nih.gov/

2
http://avogadro.cc

3
https://www.rcsb.org/
